# Lapcin, a potent dual topoisomerase I/II inhibitor discovered by soil metagenome guided total chemical synthesis

**DOI:** 10.1038/s41467-022-28292-x

**Published:** 2022-02-11

**Authors:** Zongqiang Wang, Nicholas Forelli, Yozen Hernandez, Melinda Ternei, Sean F. Brady

**Affiliations:** Laboratory of Genetically Encoded Small Molecules, The Rockefeller University, New York, NY USA

**Keywords:** Drug delivery, Metagenomics

## Abstract

In natural product discovery programs, the power of synthetic chemistry is often leveraged for the total synthesis and diversification of characterized metabolites. The synthesis of structures that are bioinformatically predicted to arise from uncharacterized biosynthetic gene clusters (BGCs) provides a means for synthetic chemistry to enter this process at an early stage. The recent identification of non-ribosomal peptides (NRPs) containing multiple ρ-aminobenzoic acids (PABAs) led us to search soil metagenomes for BGCs that polymerize PABA. Here, we use PABA-specific adenylation-domain sequences to guide the cloning of the *lap* BGC directly from soil. This BGC was predicted to encode a unique N-acylated PABA and thiazole containing structure. Chemical synthesis of this structure gave lapcin, a dual topoisomerase I/II inhibitor with nM to pM IC50s against diverse cancer cell lines. The discovery of lapcin highlights the power of coupling metagenomics, bioinformatics and total chemical synthesis to unlock the biosynthetic potential contained in even complex uncharacterized BGCs.

## Abstract (web summary)

Chemical synthesis of secondary metabolites isolated from nature, and derivatives thereof, is still a paradigm of significance to drug development. Here the authors instead use bioinformatics to analyze a biosynthetic gene cluster found in the soil metagenome, and chemical synthesis of its predict product to produce lapcin, a dual topoisomerase I/II inhibitor with promising activity against cancer cell lines.

## Introduction

Biologically active bacterial metabolites have been a principal source of inspiration for the development of diverse small molecule therapeutics^1–4^. A key role for synthetic chemistry in this discovery process is the total synthesis and synthetic derivatization of natural products that have been physically isolated and structurally characterized from bacterial fermentation broths. The focus on physically characterized structures significantly limits the use of synthetic chemistry in the study of natural chemical diversity as most natural product biosynthetic gene clusters (BGCs) are not expressed (i.e., silent) in the laboratory; and therefore the metabolites they encode remain a mystery. We believe that, in a growing number of instances, the ability to bioinformatically predict the output of a BGC has developed to the extent where the chemical synthesis of a bioinformatically predicted structure (i.e., a synthetic-Bioinformatic Natural Product or syn-BNP)^5,6^ now provides an alternative, purely in vitro method for converting the genetic information encoded in a BGC into a bioactive small molecule. The application of total synthesis methods to bioinformatically predicted small molecules provides an opportunity for synthetic chemistry to enter the natural product discovery pipeline at a much earlier phase. By focusing on the synthesis of previously inaccessible natural structures instead of already discovered natural products, synthetic chemistry could significantly expand its impact on the natural products drug discovery process.

Uncovering unexploited biosynthetic diversity is key to the identification of BGCs whose bioinformatic structure predictions can serve as appealing starting points for the total synthesis of bioactive syn-BNPs. One of the most common mechanisms by which bacteria generate biologically active small molecules is the polymerization of alpha amino acids using non-ribosomal peptide synthetases (NRPSs)^7^. The recent discovery of two structurally related antibiotics, albicidin and cystobactamid^8,9^, that arise from NRPSs that polymerize ρ-aminobenzoic acid (PABA) monomers suggests that bacteria might produce a previously undiscovered collection of bioactive metabolites using an alternative substrate polymerization strategy than has been seen in most NRPS derived natural products characterized to date. Publicly available sequenced bacterial genomes do not appear to contain PABA polymerizing BGCs beyond those that are predicted to encode albicidin or cystobactamid. As the majority of environmental bacteria are still not readily cultured in the laboratory, we postulated that PABA polymerizing BGCs might be more commonly associated with the uncultured bacteria present in the environment.

Here we track PABA specific adenylation (A) domain sequences in soil metagenomic libraries and find that NRPS BGCs that are predicted to utilize PABA are common in these environments. Detailed bioinformatic analysis of one such BGC, the *lap* BGC, predicted that it encodes an N-acylated mixed PABA thiazole-based structure. Total chemical synthesis of the bioinformatically predicted *lap* BGC product gave a syn-BNP that we have called lapcin. Lapcin is a potent dual topoisomerase I/II inhibitor that shows low nM to pM cytotoxicity against diverse cancer cell lines and represents a distinct structural class of topoisomerase inhibitors. The discovery of lapcin represents a compelling, structurally complex, example of the potential power of linking synthetic chemistry and bioinformatics to unlock the biosynthetic instructions hidden in complex silent BGCs. Furthermore, this work shows that coupling metagenome BGC discovery methods with a syn-BNP approach provides a method for circumventing difficulties associated with both culturing bacteria and activating BGCs, two key bottlenecks that have hampered the discovery of bioactive small molecules encoded by many bacterial BGCs.

## Results

### Discovery of the lapcin BGC

**Fig. 1:**
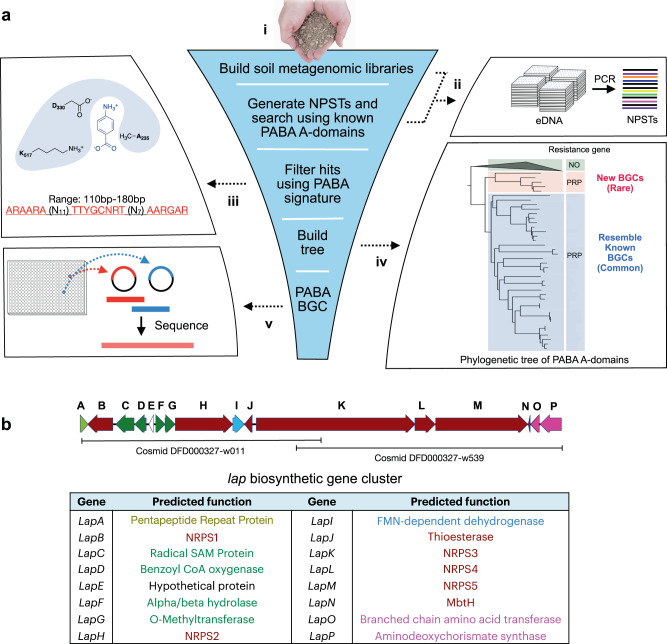
Discovery of the lap gene cluster. **a** Overview of the PABA-specific A-domain guided discovery of the lapcin (*lap*) BGC from the soil metagenome. i DNA extracted from soil was used to construct metagenomic libraries. ii NPSTs generated from arrayed metagenomic libraries and iii the resulting NPSTs were searched for PABA-specific A-domains based on a signature sequence derived from known PABA A-domains. iv Phylogenetic analysis of predicted PABA NPSTs was used to identify sequences that arose from a new family of BGCs. v Clones containing NRPS BGCs of interest were recovered from the arrayed library subpools and fully sequenced to reveal BGCs that encode PABA-based natural products. **b** The *lap* BGC, which is shown here, was recovered from an archived soil metagenome library using this process. olive, resistance gene; red, NRPS biosynthesis; green, PABA tailoring; blue, thiazole formation; pink, PABA core.

In an effort to expand the biosynthetic diversity we can interrogate for BGCs that might encode interesting bioactive natural products we have created a collection of cosmid clone libraries containing DNA extracted directly from diverse soil samples (environmental DNA, eDNA)^10,11^. In total, this collection contains almost 1 × 10^9^ ~40 kb fragments of cloned eDNA. To simplify the screening and recovery of clones containing BGCs of interest, soil eDNA libraries were divided into sub-pools of ~25,000 unique clones each. In addition, to facilitate the search for NRPS BGCs in these libraries, cosmid DNA isolated from each library sub-pool was used as the template in PCR reactions with barcoded A-domain specific degenerate primers. A-domain amplicons were sequenced and the resulting reads were clustered to generate A-domain markers (natural product sequence tags, NPSTs) for NRPS BGCs captured in each library sub-pool (Fig. 1a-i). We used a two-step screening process to identify PABA-specific A-domain NPSTs. Initially, NPSTs were compared to characterized A-domain sequences using the environmental surveyor of natural product diversity (eSNaPD) software package (Fig. 1a-ii)^12^. eSNaPD was designed to identify sequences that are more closely related to a target domain sequence than any other sequences in GenBank, suggesting a common evolutionary ancestor and therefore a common biosynthetic product. NPSTs that showed the highest sequence identity to a known PABA-specific A-domain were retained. In a second round of screening, we took advantage of sequence differences seen in PABA-specific A-domains. NPSTs identified by eSNaPD were examined for the presence of three conserved sequences found in known PABA-specific A-domains (Fig. 1a-iii), including the presence of an alanine at position 235 in place of the aspartic acid that normally interacts with the α-amino group of an amino acid^13^. NPSTs that passed both filters were considered PABA-specific NPSTs and were used to generate a phylogenetic tree to guide our discovery of previously uncharacterized PABA-containing BGCs.

**Fig. 2:**
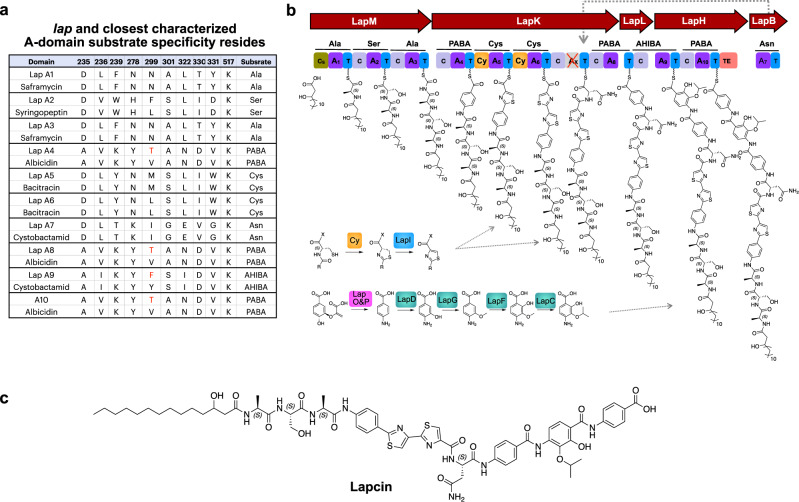
Bioinformatic prediction of lapcin from the lap gene cluster. **a** The substrate of each lapcin A-domain was inferred by comparing the 10 residues that make up the substrate binding pocket of each lapcin A-domain with characterized NRPS A-domains (red, difference between *lap* and known substrate binding pockets). **b** The structure of lapcin was predicted bioinformatically based on the NRPS modules and accessory enzymes found in the *lap* gene cluster. **c** The predicted structure of lapcin.

NPSTs that were very closely related to PABA-specific A-domains from characterized BGCs formed a clade that has representatives from many of the soils in our cosmid library (Fig. 1a-iv). In addition to this large and common clade, we identified a second smaller, soil metagenome derived clade. We predicted that NPSTs from this clade likely arose from a novel, and potentially rare, family of PABA encoding BGCs. eDNA cosmids associated with a representative NPST in this clade were recovered from the appropriate library sub-pools. Sequencing of the isolated cosmids revealed a BGC (*lap*) with five NRPS genes (*lap* b, h, k, l, m) that encode 10 modules, suggesting the production of a decapeptide (Figs. 1b and 2; Supplementary Fig. 1 and Supplementary Table 1). The edge of the *lap* BGC was defined by the appearance of genes predicted to be involved in primary, instead of secondary, metabolism (Supplementary Table 2).

### Bioinformatic prediction

The functional order of the 10 modules in lap BGC can be inferred from an analysis of domains present in each NRPS protein. Module 1 in LapM (NRPS5) contains a condensation starter (Cs) domain that is predicted to initiate peptide biosynthesis with a lipid (Supplementary Fig. 2). Almost all characterized close relatives of this Cs- domain use β-hydroxy C10 to C14 lipids (Supplementary Fig. 3). We propose that a similar lipid would be used to initiate the biosynthesis of the *lap* BGC product. The presence of the thioesterase (TE) domain at the end of LapH (NRPS2) indicates the *lap* peptide terminates at this module. The domain content of the terminal modules in the *lap* NRPS proteins allow us to place LapK (NRPS3) and LapL (NRPS4) between the initiating megasynthetase LapM and the terminal megasynthetase LapH. The substrate binding pocket in the penultimate A-domain of LapK is missing key conserved residues, suggesting that it is not active (Supplementary Fig. 4)^8,9,14^. As is seen in other BGCs with inactive A-domains, including other PABA-encoding BGCs, the isolated A-domain in LapB (NRPS1) was predicted to function in trans with *LapK*^8,14^.

The substrate specificity of each NRPS module was predicted based on the 10 amino acids that makeup an A-domain substrate binding pocket^13^. Each *lap* A-domain code has an identical, or nearly identical, match among A-domains from characterized BGCs, thus allowing for a high confidence substrate prediction to be made for each A-domain (Fig. 2a). The only disagreements were at position 299 which is known to be the most variable position in the A-domain substrate binding pocket^13^. Four A-domains (AD4, AD8, AD9, AD10) were predicted to use PABA-like substrates. ADs 4, 8 and 10 were predicted to be specific for PABA, while AD9 was predicted to use the modified PABA substrate, 4-amino-2-hydroxy-3-isopropoxybenzoic acid (AHIBA). The use of AHIBA by at least one A-domain is supported by the presence of *lap* D, F, G, and C, which were predicted to encode for the hydroxylation, methylation and isomethylation of PABA (Fig. 2b). Two modules were predicted to encode cysteine-specific A-domains (AD5 and AD6). Both of these modules contain heterocyclization condensation (Cy) domains, suggesting the formation of two thiazoline rings. Furthermore, the presence of a predicted FMN-dependent dehydrogenase (LapI), suggested that ultimately the two cysteines are converted to two thiazole rings. The remaining 4 modules were predicted to introduce 4 additional proteinogenic amino acids: Ala (AD1), Ser (AD2), Ala (AD3), Asn (AD7). In the case of cystobactamid and albicidin, LapB homologs that install Asn-7, together with other tailoring enzymes that are not encoded by the *lap* BGC, are thought to be responsible for generating a number of different L-asparagine modifications^14^. As the naturally produced collections of cystobactamid and albicidin both include simple L-asparagine containing congeners that show potent activity, we included L-asparagine in our structure prediction of lapcin^15,16^.

Taken together, this analysis allowed us to predict the product of the *lap* BGC as an N-acylated decapeptide containing two thiazoles, four PABAs and four proteinogenic amino acids. We have called this structure lapcin. While the right-hand tri-PABA substructure is similar to that seen in the antibiotics albicidin and cystobactamid, the majority of the structure is completely distinct from previously characterized natural products (Supplementary Table 3). In fact, no N-acylated or thiazole containing NPRS-derived PABA-based natural products have been identified in traditional natural product screening programs.

As the *lap* BGC was cloned directly from the complex mixture of bacteria present in a soil metagenome, the exact organism from which it was cloned is not known. A BLAST search indicated that the closest relative of each individual gene found in the *lap* gene cluster most often arose from the genome of a myxobacterium (Supplementary Table 1). Cultured Myxobacteria are often rich in secondary metabolite BGCs. Unfortunately, most members of this group of bacteria are believed to remain uncultured^17–19^. Direct cloning of DNA from environmental samples as we have done here circumvents this culture bottleneck; however, it introduces the challenge of accessing metabolites encoded by captured BGCs. As our understanding of natural product biosynthesis has matured, it has become possible to make increasingly accurate predictions about the structure encoded by a BGC. Our analysis of the *lap* BGC suggested that lapcin was likely an accurate representation of the intended product of this BGC and that total chemical synthesis was therefore a viable method for accessing the metabolite, or at least a close analog of the metabolite encoded by the *lap* BGC. To be successful, a syn-BNP does not need to be a perfect copy of a natural product, only an analog that is close enough to mimic its natural biological activity.

### Total chemical synthesis

**Fig. 3:**
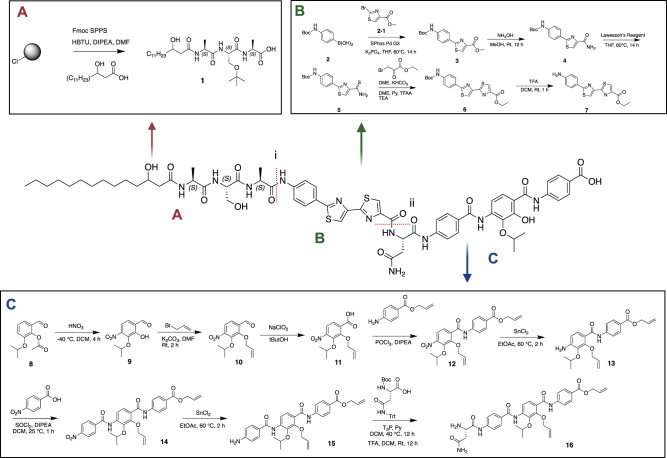
Overview of the synthesis of lapcin. Lapcin was synthesized in three fragments **A**–**C** which were coupled using a series of amid bond forming reactions. i Coupling fragment AB (**S18**): isobutyl chloroformate, TEA, THF. ii Coupling AB (**S18**) to fragment C (**16**): HBTU, DIPEA, DMF.

Our retrosynthetic analysis of lapcin suggested two amide bond disconnections to give 3 fragments (A-C) that could be readily synthesized and coupled (Fig. 3). Firstly, the preparation of fragment A began with the synthesis of the peptide portion on 2-chlorotrityl chloride (CTC) resin using standard Fmoc-based solid-phase peptide synthesis (SPPS) methods (Fig. 3, Supplementary Fig. 5). With the tripeptide complete, DL-3-hydroxy myristic acid was appended to its N-terminus. NRPS derived lipopeptides are often found naturally as mixtures with different fatty acids. As there is unlikely to be one correct answer to the exact lipid found naturally on lapcin, it was synthesized using a racemic version of one of the most frequently seen lipids in NRPS derived lipopeptides, DL-3-hydroxy myristic acid. The resulting fatty-acyl tripeptide was released from the CTC resin using 20% hexafluoroisopropanol (HFIP) to give a protected product ready for amide coupling (**1**). To obtain the two thiazole rings in fragment B (Fig. 3, Supplementary Fig. 6), 4-(N-Boc-amino)phenylboronic acid (**2**) was initially linked to ethyl 2-bromothiazole-4-carboxylate (**2-1**) using a Suzuki-Miyaura coupling^20^. After conversion to the corresponding amide (**4**) and thionation by Lawesson’s reagent, a second thiazole was installed through a Hantzch thiazole synthesis (**7**)^21^. Finally, the synthesis of fragment C (Fig. 3, Supplementary Fig. 7) was started with synthesis of the alloc-protected PABA subunits from 2-hydroxy-3-isopropoxy-4-nitrobenzaldehyde (**9**)^22^. The exposed ortho hydroxyl was alloc-protected prior to oxidation of the aldehyde to a carboxylic acid of compound **11**. A second alloc-protected PABA subunit **(11-2)** was coupled to this carboxylic acid using phosphoryl chloride (POCl_3_) and N,N-diisopropylethylamine (DIPEA). Tin(II) chloride (SnCl_2_) was used to reduce the nitro group to the free amine of compound **13**, which was then coupled to 4-nitrobenzoic acid. Subsequent nitro-reduction to compound **15** followed by solution phase coupling (T_3_P, Py) of Boc-Asn(Trt)-OH and deprotection yielded the complete fragment C (**16**). Fragments A and B were connected by activating the free carboxylic acid on A with isobutyl chloroformate. After hydrolysis to the carboxylic acid, the resulting AB (**S18**) fragment was coupled to fragment C using HBTU-mediated amide bond formation to give the desired final protected product (**S19**, Supplementary Fig. 8). After deprotection, lapcin was purified by high performance liquid chromatography (HPLC) and its structure confirmed by 1 and 2D NMR spectroscopy as well as HRMS (Supplementary Fig. 9–30).

### Heterologous expression

In addition to synthesizing lapcin, we also tried to access the metabolite encoded by the *lap* gene cluster using a biological system (Supplementary Fig. 31). The two overlapping eDNA cosmids containing the *lap* BGC were assembled into a contiguous fragment of DNA using transformation association recombination (TAR) in yeast. The TAR assembly reaction was carried out using pTARa4, a broad host range yeast artificial chromosome (YAC): bacterial artificial chromosome (BAC) shuttle vector that is capable of being introduced into a wide range of bacterial taxa. For heterologous expression purposes, the *lap* BGC-containing BAC, pTARa4-lap, was either electroporated or conjugated into *Myxococcus xanthus* DK1622, *Streptomyces albus J1074, Streptomyces coelicolor M1152*, and *Pseudomonas putida KT2440*^23,24^. Culture broth extracts from strains transformed with either pTARa4-lap or the empty pTARa4 shuttle vector were compared by high resolution liquid chromatography mass spectrometry (HR-LCMS) to look for *lap* BGC-specific metabolites. Unfortunately, none of these strains, even when grown under multiple culture conditions, produced any detectable *lap* BGC-specific metabolites. This was not particularly surprising in light of the fact that most natural product BGCs are silent in the laboratory and that is the key reason for exploring a syn-BNP approach for accessing bioactive small molecules from the genetic instructions contained in bacterial BGCs.

### Biological activity and model of action

**Fig. 4:**
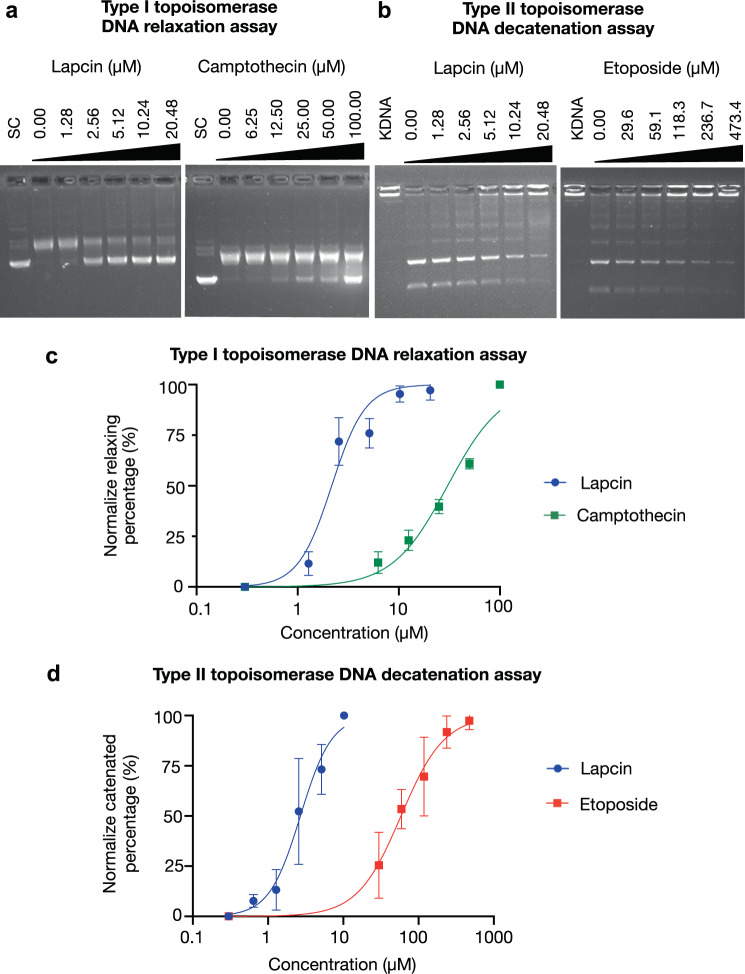
Topoisomerase inhibition by lapcin. **a** Type I topoisomerase DNA relaxation assay (SC, supercoil plasmid) **b** Type II topoisomerase DNA decatenation assay (KDNA, Kineotoplast DNA). **c** Type I and **d** II topoisomerase inhibition plots. *n* = 3 independent assays. Mean value and SD are shown.

As an initial step to assess lapcin’s bioactivity, we assayed lapcin for toxicity against diverse microbial pathogens and human cancer cell lines. At the highest concentration we tested, lapcin showed no antimicrobial or antifungal activity (Supplementary Table 4). It was, however, found to be a potent human cancer cell line toxin (Table 1, Supplementary Table 5, Supplementary Figs. 32–35). Previously reported NRPS-derived PABA-based natural products (i.e., albicidin and cystobactamid) are polymer antibiotics that inhibit the bacterial DNA gyrase^9,25^. Self-resistance to these antibiotics is provided by a pentapeptide repeat protein (PRP) encoded in the producing BGC^8,9,26^. PRPs are a large class of proteins with conserved, tandemly repeated amino acids. The function of most PRPs is unknown; however, one role that has been assigned to them is protection of topoisomerases against small molecule toxins^27,28^. Small molecules encoded by BGCs that contain PRP genes have been shown to have activity against both prokaryotic and eukaryotic topoisomerases^26^ The gene directly adjacent to *lapB* (NRPS1) was predicted to encode a PRP, LapA (Fig. 1b), suggesting that lapcin might also be a topoisomerase inhibitor. To explore this hypothesis, we tested the activity of lapcin against bacterial DNA gyrase as well as the two major human topoisomerases (Topo I and II) using in vitro DNA relaxation (DNA gyrase, Topo I) and decatenation (Topo II) assays. Lapcin showed weak activity against DNA gyrase (IC50 > 20.5 μM, Supplementary Fig. 36), which likely explains its lack of antibacterial activity when applied extracellularly. The *lapA* gene may be retained in the lap BGC to provide protection against low level bacterial DNA gyrase activity while the natural product remains in the cell of the producing bacterium. While only weakly active against DNA gyrase, lapcin was a potent of inhibitor of both DNA relaxation by topoisomerase I (IC50 2.17 μM) and DNA decatenation by topoisomerase II (IC50 7.53 μM) (Fig. 4, Supplementary Fig. 37). This is 14 times more potent than the alkaloid camptothecin upon which a number of clinically used topoisomerase I inhibitors are based (IC50 30.4 μM), and almost 15 times more potent than the clinically used topoisomerase II inhibitor etoposide (IC50 108 μM) (Fig. 4). Compounds that block topoisomerases either inhibit the catalytic activity of the enzyme (an inhibitor) or increase the level of topoisomerase-mediated DNA cleavage (a poison). When we examined lapcin’s activity in topoisomerase I and II DNA cleavage assays, we did not observe the accumulation of a cleavage product in either assay (e.g., nick DNA in Topo I assay or linear DNA in Topo II assay), suggesting enzyme inhibition as its mechanism of action. Topoisomerase inhibitors are a mixed group of compounds that either intercalate DNA thereby interfering with the binding between DNA and topoisomerase or disrupt topoisomerase catalytic activity by binding the enzyme itself. As intercalators interact with the DNA substrate and not the enzyme their inhibitory activity is largely independent of enzyme concentration. Unlike known DNA intercalators, lapcin’s activity showed a strong dependence on enzyme concentration in both Topo I and II activity assays (Supplementary Fig. 38). Taken together these data indicate lapcin is a dual Type I and II topoisomerase catalytic inhibitor that inhibit the catalytic activity of both enzymes.

**Table 1:** Lapcin activity against human cell lines.

Cancer type	IC50 (nM)		
Cell line	Lapcin	Etoposide	Camptothecin
Colon cancer			
HT29	0.516	9,610	22.3
Colo205	0.504	>54,400	95.7
HCT116	923	>54,400	129
SW480	37.2	26,900	284
Breast cancer			
MCF 7	35.1	333	>29,200
HCC1806	212	3,330	19.6
Lung cancer			
A549	2.66	79.9	17.6
NCI-H1299	0.0168	716	15.0
NCI-H226	241	217	28.5
Other			
Hela (Cervical)	0.986	1,510	62.6
U2OS (Bone)	1.29	1,840	172
Normal cell			
HEK293	48.4	729	19.2

*Note*: IC50s were rounded to three significant figures. *n* = 3 biologically independent cells.

Lapcin was a nM to pM (IC50) inhibitor of the cell lines we tested. These included breast, lung, colon, bone and cervical cancer cell lines. Lapcin was more potent than the topoisomerase II inhibitor etoposide against all of the cell lines we tested, with the exception of the lung cancer cell NCI-H226, against which it was essentially equipotent. Similarly, with the exception of a few cell lines (HCT116, HCC1806 and NCI-H226) lapcin was more potent than the topoisomerase I inhibitor camptothecin. Consistent with its in vitro topoisomerase inhibition, we saw a general correlation between lapcin activity and reported p53 expression levels in the cancer cell lines we tested^29–31^. Cell lines expressing wild-type p53 (HCT116, H226, MCF7) tended to show higher IC50s than p53 reduced cell lines (NCI-H1299, HT29, Colo205, Hela)^29,32–34^. This correlation was not perfect; for example, the breast cancer cell line HCC1806 is reported to express negligible p53 and the non-small cell lung cancer cell line A549 is reported to express wild type p53^32^. Lapcin was most active (IC50 16.8 pM) against NCI-H1299, a non-small cell lung cancer cell line that lacks the expression of p53 protein. In the case of the human bone osteosarcoma epithelial cell line U2OS, both anticancer agents we tested as controls showed elevated IC50s, while lapcin retained potent activity.

## Discussion

Sequencing of A-domain PCR amplicons from soil metagenomic libraries identified a number of sequences that we predicted arose from BGCs that use PABA monomers instead of alpha amino acids. The cloning and sequencing of one such BGC revealed the *lap* BGC, which we bioinformatically predicted would encode a *N*-acylated thiazole and PABA containing decapeptide, lapcin. To circumvent the challenge of decoding the *lap* BGC using biological processes, we produced lapcin by total chemical synthesis. Lapcin is a dual topoisomerase I/II inhibitor that inhibits the growth of diverse cancer cells. Topoisomerase inhibitors are clinically validated targets for anticancer therapy^35^. While there are currently no topoisomerase inhibitors in clinical use, topoisomerase poisons are used in the treatment of a number of cancers including, breast, lung, testicular, and prostate, with a number of additional candidates in clinical trials^36,37^. Lapcin is structurally distinct from any previously identified topoisomerase inhibitor (including poisons and catalytic inhibitors), providing a different structural class to investigate as antineoplastic agents. Between the non-cancerous HEK293 cell line control and most susceptible cell lines we tested, there is over 3 orders of magnitude difference in IC50 (Table 1), suggesting that lapcin has a sufficient therapeutic window to explore its utility as an antineoplastic agent in vivo. To the best of our knowledge, lapcin is the first NPRS derived *N*-acylated or thiazole containing PABA-based natural product.

The number of uncharacterized sequenced BGCs, whether derived from cultured bacteria or metagenomes, is rapidly increasing. Unfortunately, the rate at which the instructions contained in these BGCs are converted into chemical entities remains very low. Our discovery of lapcin confirms that a syn-BNP approach represents a viable alternative strategy for generating even complex classes of biomedically relevant molecules from uncharacterized BGCs. Traditional natural product total synthesis efforts have almost exclusively focused on characterized bioactive natural products. Syn-BNP methods provide a alternative paradigm where targets for total synthesis are structures that are bioinformatically predicted from silent BGCs. Lapcin provides a key proof of principle example of a syn-BNP approach being able to generate a previously unknown small molecule whose potency rivals that of natural products produced biologically.

## Methods

### Identification of the *lap* biosynthetic gene cluster in the soil metagenome

Archived eDNA cosmid libraries were used to screen for BGCs that encode PABA containing natural products. Procedures for library construction and A-domain screening to facilitate BGC discovery have been described in detail previously^10,38,39^. Briefly, crude environmental DNA (eDNA) was obtained from ~0.5 kg of soil by heating (70 °C) in lysis buffer (100 mM Tris-HCl, 100 mM ethylenediaminetetraacetic acid, 1.5 M NaCl, 1% (w/v) hexadecyltrimethylammonium bromide, 2% (w/v) sodium dodecyl sulfate, pH 8.0) for 2 h. Soil particulates were removed from the crude lysate by centrifugation, and eDNA was precipitated from the resulting supernatant with the addition of 0.7 volumes isopropanol. Crude eDNA was collected by centrifugation, washed with 70% ethanol and resuspended in TE buffer. Crude eDNA was purified by preparative agarose gel electrophoresis to yield pure high-molecular-weight (HMW) eDNA. HMW eDNA was blunt ended (Epicentre, End-It), ligated into pWEB-TNC, packaged into lambda phage and transfected into *Escherichia coli* EC100. Following recovery, transfected cells were selected using chloramphenicol (12.5 μg/mL). The resulting clones were arrayed at a density of ~25,000 clones per pool. Matching glycerol stocks and cosmid DNA minipreps were prepared from each pool. Pool-specific barcoded A-domain degenerate primers (AD-FW: 5′-GCSTACSYSATSTACACSTCSGG-3′ and AD-RV: 5′-SASGTCVCCSGTSCGGTA-3′) were used to amplify A-domain sequences from each library sub-pool. These primers were designed to recognize the conserved A3 and A7 regions in NRPS A-domains^38–40^. PCR reactions: 12 μl reaction, 1× G buffer (Epicentre), 50 pmol of each primer, 2.5 Omni Klentaq polymerase (DNA Polymerase Technology) and 100 ng eDNA. Cycle conditions for AD amplification: 95 °C 4 min, (95 °C 30 s, 63.5 °C 30 s, 72 °C 45 s) × 34 cycles, 72 °C 5 min. Prior to sequencing all PCR amplicons were quantified by gel electrophoresis and mixed in an equal molar ratio. The resulting pool was fluorometrically quantified with a HS D1000 ScreenTape (Agilent Technologies) and sequenced using the Illumina MiSeq Sequencing System technology.

The resulting reads were de-barcoded, trimmed and clustered at 95% using UCLUSTER^41^ to generate NPSTs. The eSNaPD^12^ software package was used to identify NPSTs that were most closely related to PABA specific A-domain known BGCs (albicidin and cystobactamid). In particular, NPSTs that returned an E-value of less than 10^−25^ to a known PABA specific A-domain were considered primary hits. Primary hits were screened for the following conserved 47 base pair sequence that is unique to known PABA specific A-domains: (ARAARA (N11) TTYGCNRT (N7) AARGAR, Y = C/T; R = A/G; N = A/T/C/G). NPSTs that passed this filter were considered to be associated with potential PABA specific A-domains. Hit sequences were aligned by MUSCLE algorithm using Macvector 18.0.2^42^. The phylogenetic tree used to guide the discovery of the *lap* BGC was visualized using iTOLv5 software^43^. NPSTs associated with clades that did not contain any known PABA A-domains were assumed to arise from BGCs that encode previously uncharacterized families of PABA containing natural products. To identify the *lap* BGC, two overlapping eDNA clones (DFD000327-539 and DFD000327-11) associated with one such PABA specific A-domain clade were recovered from distinct sub-pools of an archived metagenomic library using a previously described dilution PCR method^38^. These clones were sequenced using Illumina MiSeq technology. A single continuous eDNA contig containing the *lap* BGC was assembled from this data using Newbler 2.6 (Roche).

In silico analysis of *lap* BGC. The *lap* BGC was annotated using a pipeline consisting of open reading frame (ORF) prediction and BLAST searches. To predict the amino acid specificity of each A-domain sequence in the *lap* BGC, the sequence was analyzed using the online version of antismash v5.1.2 (bacterial)^44^. The 10 amino acids (positions 235, 236, 239, 278, 299, 301, 322, 330, 331, 517) making up each A-domain substrate binding site were compared to the corresponding amino acids from A-domains found in characterized natural product BGCs to predict the substrate of each *lap* A-domain^13^. This information combined with the predicted functions of the tailoring enzymes found in the *lap* BGC was used to determine the final structure of lapcin.

### Cell viability assay

An MTT (2-(4,5-dimethylthiazol-2-yl)−2,5-diphenyltetrazolium bromide) assay was used to determine the cytotoxicity of lapcin towards diverse cancer cell lines^45^. Lapcin was dissolved in DMSO to make a 3.2 mg/mL working solution. For each cancer line detailed in Supplementary Table 5, cells at 80-90% confluency were counted and seeded in 96-well, flat bottom microplates and incubated at 37 °C with a 5% CO_2_ atmosphere. Outer wells were unused to avoid edge effects. After adhering for 24 h, the medium was sterilely aspirated and replaced with 100 µL of fresh medium containing lapcin or a control compound serially diluted at concentrations ranging from 8,000 to 0.00382 ng/mL. After 48 h (37 °C, 5% CO_2_), the medium was carefully removed and 110 µL of freshly prepared MTT solution (10 µL of 5 mg/mL MTT in PBS (pH 7.4) premixed with 100 µL of complete medium) was added to each well. After 3 h at 37 °C with 5% CO_2_, 100 µL of solubilization solution (40% DMF, 16% SDS and 2% acetic acid in H_2_O) was added to each well and precipitated formazan crystals were allowed to dissolve for 4 h. The absorbance of each well was then measured at 570 nm using a Tecan microplate reader. IC50 values were calculated as the concentration of each compound required for 50% inhibition of cell growth relative to the no compound controls (Graphpad Prism 9.0).

## Supplementary information

Supplementary information

## Data Availability

All the characterization data and experimental protocols are provided in the article and its supplementary information. The *lap* BGC has been annotated and deposited in the NCBI database under deposition number MZ165589.
