# Supplementary material for: Lapcin, a potent dual topoisomerase I/II inhibitor discovered by soil metagenome guided total chemical synthesis
Source: Nat Commun. 2022 Feb 11;13:842. doi: 10.1038/s41467-022-28292-x (PMC8837603; doi:10.1038/s41467-022-28292-x)
Supplement: Supplementary file 1 — Supplementary information [file 41467_2022_28292_MOESM1_ESM.docx]

**Supplementary Information:**

**Title:** Lapcin, a potent dual topoisomerase I/II inhibitor discovered by soil metagenome biosynthetic gene cluster guided total chemical synthesis.

**Authors:** Zongqiang Wang‡, Nicholas Forelli‡, Yozen Hernandez, Melinda Ternei and Sean F. Brady*

**Affiliation**: Laboratory of Genetically Encoded Small Molecules, The Rockefeller University, 1230 York Avenue, New York, NY 10065

‡ These authors contributed equally to this work.

**Corresponding Author:** Sean F. Brady

**Contact**: Laboratory of Genetically Encoded Small Molecules

The Rockefeller University

1230 York Avenue

New York, NY 10065

**Phone:** 212-327-8280

**Fax:** 212-327-8281

**Email:** [sbrady@rockefeller.edu](mailto:sbrady@rockefeller.edu)

**1. Supplementary Methods**

**General experimental procedures and materials**

Unless stated otherwise, all reactions conducted in organic solvents were performed in oven-dried glassware under an atmosphere of argon or nitrogen. All solvents and reagents were purchased from commercial sources and used without further purification. Flash column chromatography was performed using a CombiFlash Rf 200 system equipped with a normal phase RediSep Rf silica gel column. Semipreparative HPLC was performed on an Agilent 1200 Series HPLC equipped with an XBridge Prep C18 130 Å column (10 x 150 mm, 5 µm) and a Shimadzu LC-20AB HPLC equipped with a Phenomenex C18 column (40 x 150 mm) or Phenomenex C18 column (30 x 75 mm). HRMS data were acquired on a SCIEX ExionLC UPLC coupled to an X500R QTOF mass spectrometer, equipped with a Phenomonex Kinetex PS C18 100 Å column (2.1 x 50 mm, 2.6 µm) and operated by SCIEXOS software. ^1^H NMR and ^13^C NMR spectra were acquired at room temperature on a Bruker AV600 or Bruker Avance NEO operating at 600 or 400 MHz. Chemical shift values were reported in ppm and referenced to residual solvent signals, for ^1^H NMR: DMSO-*d_6_* = 2.50 ppm; CDCl_3_ = 7.26 ppm; MeOD-d*_4_* = 3.31 ppm for ^13^C NMR: DMSO-*d_6_* = 39.5 ppm; CDCl_3_ = 77.1 ppm; MeOD-d*_4_* = 49.2 ppm.

**Clone Recovery of lapcin BGC from eDNA libraries:** The well locations of cosmids of interest were identified using well-specific barcode sequences incorporated in the A-domain degenerate primers. A serial dilution PCR method was used to recover the single clone of cosmid.^1^ Target library wells containing 1 of ~25,000 unique cosmids were inoculate into 3 mL LB containing 12.5 μg/mL chloramphenicol and 100 μg/mL carbenicillin and were then grown overnight at 37℃. The saturated O/N culture was then diluted in LB medium to a concentration of 3000 CFU/mL as judged by OD 600_nm_. 50 uL of the dilution was then transferred into 384-well plates to a concentration of ~500 CFU/well and then grown to confluence. Real-time PCR was used to screen and identify wells containing target clones. This was repeated to identify wells containing targets as 1 of ~5 clones. Five clone pools were then plated on LB agar medium (contain the same antibiotics), and target clones were identified by colony PCR. Recovered single clones containing lapcin encoding sequences were mini-prepped and sequenced by Illumina MiSeq technology. To identify the overlapping cosmid, two specific overlapping primers targeting the edge (1 kb region) and middle (10 kb region) of the primary cosmid was designed manually and screened against cosmid DNA pools. The hits that match to the 1 kb edge region but not the 10 kb middle region will be recognized as an overlapping clone and recovered to a single clone. The primer sequences are listed in **Supplementary Table 6**.

**TAR assembly of *lap* BGC from overlapping cosmids**

To facilitate heterologous expression studies the shuttle vector pTARa4 was constructed by inserting a synthetic IR-TPs cassette (IR, inverted repeat; Tps, MycoMar transposase gene: DQ236098) and a kanamycin resistance gene into the SphI site of the pTARa-lysis shuttle vector.^2,3^ Yeast based transformation association recombination (TAR) was used to assemble overlapping cosmids, DFD00327-539 and DFD000327-11 and pTARa4 into a *lap* BGC containing bacterial artificial chromosome (BAC). Detailed procedures for the assembly of overlapping cosmid clones have been described previously.^2,4^ Briefly, specific primers designed to target the distal ends of the two overlapping cosmids were used to generate two ~500 bp amplicons with overlapping regions needed for their directional cloning into pTARa4. The PCR products were then seamless merged into linearized pTARa4 vector (pre-digested by PacI) using Gibson assembly (NEB, USA) to give a lapcin BGC capture vector. 100 ng of the PmeI-cut lapcin BGC capture vector and 200 ng of each DraI-cut overlapping cosmid were co-transformed into 200 μL of *Saccharomyces cerevisiae* CRY1-2 spheroplasts.^4^ Transformed spheroplasts were overlaid onto SC-lys dropout agar plates and incubated at 30 °C until colonies appeared (∼72 h). Yeast colonies were screened for the correct assembly of the two overlapping clones using primer pairs spaced at 10 kb intervals across the *lap* BGC. The BAC from a yeast colony that both tested positive for all primer pairs and produced the correct predicted restriction map (Hind III digestion) was transformed into *E. coli* EPI300 (Epicenter). Miseq (illumine, USA) sequencing of this BAC (pTARa4-*lap*) confirmed its correct assembly.

**Heterologous expression of lapcin gene cluster**

pTARa4-*lap* and an empty pTARa4 vector control were individually transformed into each target heterologous expression hosts using either triparental conjugation (for *Streptomyces albus* J1074, *Streptomyces coelicolor* M1152, and *Pseudomonas putida* KT2440)^5,6^ or electro-transformation (for *Myxococcus xanthus* DK1622)^3^. For each host two pTARa4-*lap* containing clones as well as the empty control were inoculated in 50 mL starter cultures at 30 °C for 48 h. Trypticase soy broth (TSB) was used as starter culture medium for *S. albus* and *S. coelicolor*. CTT (1% casitone, 8 mM MgSO_4_, 10 mM Tris-HCl, pH 7.6, and 1 mM KH_2_PO_4_, pH 7.6) and LB (10 g/L peptone, 5 g/L Yeast Extract, 5 g/L Sodium Chloride) were used as starter culture medium for *M. xanthus* and *P. putida*, respectively. 500 μL of each start culture was then inoculated into 50 mL of fermentation medium and shaken at 30 °C for 14 days (*S. albus and S. coelicolor*) or 7 days (*M. xanthus and P. putida*). R5a broth (100 g/L sucrose, 10 g/L D-glucose, 5 g/L yeast extract, 10.12 g/L MgCl_2_·6H_2_O, 0.25 g/ L K_2_SO_4_, 0.1 g/L casamino acids, 21 g/L MOPS, 2 g/L NaOH, 5.88 mg/L CaCl_2_,80 μg/L ZnCl_2_, 400 μg/L FeCl_3_·6H_2_O, 20 μg/L MnCl_2_, 20 μg/L CuCl_2_, 20 μg/L Na_2_B_4_O_7_·10H_2_O, 20 μg/L (NH_4_)_6_Mo_7_O_24_· 4H_2_O, pH = 6.85) was used in the fermentation of *S. albus* and *S. coelicolor*. CTT broth and LB broth was used for *M. xanthus* and *P. putida,* respectively. Diaion HP20 absorbed resin (Sigma, USA) was used to absorb the secondary metabolites produced by each culture. The resin was washed with water and then bound metabolites were eluted with methanol. This eluent was analyzed on UPLC-MS system (Waters, USA) using a gradient of 5 to 95% H_2_O/acetonitrile plus 0.1% formic acid over 12 min.

**Bacteria susceptivity assay**

Lapcin was tested for antimicrobial activity against the bacteria and fungi shown in **Supplementary Table 4**. MIC assays were conducted following the protocol recommended by the Clinical and Laboratory Standards Institute.^7^ MIC assays were performed in duplicate in 96-well microplates (n = 2). Ciprofloxacin (Sigma, USA) and Nystatin (Sigma, USA) were used as positive control for bacteria and fungi, respectively. Lapcin was dissolved in sterile DMSO to make a 3.2 mg/mL working solution. Lapcin was diluted across 96-well plates using a 2-fold serial dilution method to give a concentration range of 64 to 0.06 µg/mL in 50 µL of LB broth. A single colony of each test organism was grown in either 5 mL LB (bacteria) or YPD (fungi) medium overnight at 37 °C or 30 °C, respectively. Saturated overnight cultures were diluted 5,000-fold, and then 50 µL of dilute cells were transferred into each well of the assay plates. Finally, each well contains a total volume of 100 µL. MIC values were determined by visual inspection of the minimum concentration that prevented growth after 16 h static incubation at 37 °C.

**Human Topoisomerase I DNA relaxation assay**

A commercial Human Topoisomerase I relaxation kit (Inspiralis, UK) was used to determine half-inhibitory concentration (IC_50_) of lapcin against topoisomerase I. Camptothecin (Sigma, USA) was used as the positive control and supercoiled pBR322 (Inspiralis, UK) was used as the DNA substrate in this assay. Reaction mixtures were prepared on ice and consisted of 3 µL of 10 x assay buffer (20 mM Tris-HCl pH7.5, 1mM DTT, 1mM EDTA, 50% (v/v) glycerol, 100 µg/ml albumin), 0.5 µL of 1 µg/µL pBR322. 5 µL of serial diluted lapcin or camptothecin was then added. The total reaction volume was adjusted to 29 µL with water. Finally, 1 µL of freshly diluted topoisomerase I (minimum concentration to transfer 500 ng of supercoil pBR322 to relax DNA) was added to each ice cold reaction tube. The tubes were incubated for 30 min at 37 °C and stopped by adding 30 µL of STEB (40% (w/v) sucrose, 100 mM Tris-HCl pH8.0, 10 mM EDTA, 0.5 mg/ml Bromophenol Blue) and 30 µL of chloroform/isoamyl alcohol (v:v, 24:1). The mixture was vortexed briefly and centrifuged for 2 min. 20 µL of the water phase was loaded onto a 1% (w/v) agarose gel and run at 140 v for 1 h in 0.5% TBE buffer. The gel was stained with ethidium bromide (1µg/mL in water) for 15 min and distained for 10 min in water. The gel was then visualized with a transilluminator. Image analysis (intensity of the supercoiled (SC) plasmid bands) was performed using Analytik Jena image software. All values were normalized to the negative control wells. The intensity of supercoil band was used in the determination of the IC_50_ of tested compounds and the curve was drawn in Prism 9 (9.0.0). All experiments were performed in triplicate (n = 3).

**Human Topoisomerase II DNA decatenation assay**

The decatenation assay was conducted using a Human Topo II decatenation kit (TopoGen, USA). Etoposide (Sigma, USA) was used as the positive control and kinetoplast DNA (kDNA, TopoGen, USA) was used as substrate in this assay. Assays were performed according to the manufacturer’s protocol. Briefly, on ice, 19 µL of master mixture was prepared by adding 4 µL of freshly prepared 5 x assay buffer (0.5 M Tris-HCl pH8.0, 1.50 M NaCl, 100 mM MgCl_2_, 5 mM Dithiothreitol, 300 µg/mL BSA, 20 mM ATP), 1 µL of 200 ng/µL kDNA and 5 µL of serial diluted assay compounds in 200 µL reaction tubes. 1 µL of fresh diluted topoisomerase II (1U) was then added to each reaction tube. The total reaction volume was adjusted to 20 µL with H_2_O. These reactions were incubated at 37 °C for 30 min and stopped by addition of 4 µL of 5 x Stop Buffer (TopoGen, USA). Samples were loaded directly onto a 1% (w/v) agarose gel containing 0.5 µg/ml ethidium bromide and separated at 140 v for 1 hour in 0.5% TBE buffer. The intensity of released (decatenated) circular rings was quantified using Analytik Jena image software. All values were normalized to the negative control wells. The intensity of decatenated band was used in the determination of IC50 of tested compounds and the curve was drawn in Prism 9 (9.0.0). All experiments were performed in triplicate (n = 3).

**DNA gyrase supercoiling assay**

The DNA gyrase supercoiling assay was conducted with an *E. coli* gyrase supercoiling kit (TopoGen, USA) in this assay. Ciprofloxacin (Sigma, USA) was used as the positive control. Assay was performed according to the manufacture’s protocol. Briefly, 1 µL of pHOT1 relaxed DNA (250 ng) ,4 µL of 5 x assay buffer (35 mM Tris-HCl PH 7.5, 24 mM KCl, 4 mM MgCl_2_, 2 mM dithiothreitol, 1.8 mM spermidine, 1 mM ATP, 6.5% glycerol, 0.1 mg/mL BSA) and 4 uL of serial diluted lapcin or ciprofloxacin was mixed in the reaction tube on the ice. The final volume of the reaction was adjusted to 19 µL with H_2_O. Finally, 1 µL of fresh diluted *E. coli* gyrase (1U) was added into the reaction mixture and incubated at 37 °C for 60 min. The reaction was terminated by addition of 2 µL of 10% SDS and 2 µL of 50 µg/mL proteinase K followed by a 30 min at 37 °C. The reaction was then extracted with 20 µL of chloroform/isoamyl alcohol (v:v, 24:1). The aqueous material loaded a on 1% (w/v) agarose gel and separated at 140 v for 1 hour in 0.5% TBE buffer. The gel was visualized using ethidium bromide staining. The intensity of SC plasmid bands was quantified using Analytik Jena image software. The intensity of SC bands was used in the determination of IC50 of tested compounds and the curve were drawn in Prism 9 (9.0.0). The assay was performed in triplicate (n = 3).

**Topoisomerase I/II cleavage assay:** A commercial Human topoisomerase cleavage assay kit (Inspiralis, UK) was used in this study with a slight modification. Camptothecin (Sigma, USA) and Etoposide (Sigma, USA) were used as the positive control for topoisomerase I cleavage assay and topoisomerase II cleavage assay, respectively. The assay was performed according to the manufacture’s protocol. Briefly, 29 µL of the master mixture was prepared by adding 3 µL of 10 x assay buffer, 1 µL of 1 µg/µL supercoil pBR322, 1 µL of 30 mM ATP (only used in topoisomerase II cleavage assay), 1.5 µL serial diluted compounds (dissolved in DMSO) and H_2_O. Finally, 1 µL of 10 U/µL human topoisomerase I/II alpha was added to the reaction mixture. The reaction was incubated for 30 minutes at 37℃. After incubation, 3 µL of 2% (w/v) SDS and 1.5 µL of 10 mg/mL proteinase K was added. The reaction was mixed briefly and incubated for another 30 minutes at 37 ℃. The reaction was stopped by added 10 µL of H_2_O, 40 µL of STEB stop buffer and 40 µL of Chloroform/isoamyl alcohol (v:v, 24:1). The mixture was vortexed vigorously for 10 secs and centrifuged for 2 minutes. 20 µL of aqueous phase was loaded onto 1% (w/v) agarose gel and separated at 80 V for 2 hours in 0.5% TBE buffer. The gel was stained with ethidium bromide (1 µg/mL in water) for 15 min and distained for 10 min in water and visualized using Analytik Jena image software.

**DNA intercalator assay:** A commercial DNA unwinding assay kit (Inspiralis, UK) was used in this study with a slight modification. Ethidium bromide was used as intercalator positive control. The assay contains two steps: the first step is used to determine the minimum concentration of compounds that will inhibit the enzyme activity; the second step is used to determine whether compounds act as intercalator or inhibitor. Ethidium bromide was used as the intercalator positive control and supercoil pBR322 was used as the substrate. The assay mixture was set up on the ice. Briefly, the master mixture was prepared by adding 3 µL of 2 x wheat germ topoisomerase I assay buffer, 0.5 µL of 1 µg/µL supercoil pBR322, 1.5 uL serial diluted compounds (dissolved in DMSO). H_2_O was added to a final volume of 29 µL. Finally, 1 µL of 2 U/µL Wheat germ Topoisomerase I was added to the reaction mixture. The reaction was incubated for 30 minutes at 37℃. The reaction was stopped by added 20 µL of H_2_O, and 50 µL of butanol (water-saturated). The mixture was vortexed vigorously and centrifuged for 1 minute. The lower, aqueous layer was removed and added into a new tubes containing 50 µL STEB buffer and 40 µL of Chloroform/isoamyl alcohol (v:v, 24:1). The mixture was vortexed vigorously for 10 secs and centrifuge for 2 minutes. 20 µL of aqueous phase was loaded onto 1% (w/v) agarose gel and separated at 80 V for 4 hours in 0.5% TBE buffer. The gel was stained with ethidium bromide (1 µg/mL in water) for 15 min and distained for 10 min in water and visualized using Analytik Jena image software.

**Detailed synthetic procedures**

*O*-(*tert*-butyl)-*N*-((3-hydroxytetradecanoyl)-*L*-alanyl)-*L*-seryl-*L*-alanine (**1**)

**Resin preparation**: To the 1-chloro-2-[chloro(diphenyl)methyl] benzene (6.07 g, 6.80 mmol, 1.00 eq) (1.12 g/ mmol) was added Fmoc-Ala-OH (2.12 g, 6.80 mmol, 1.00 eq) and DIPEA (27.2 mmol, 12.0 mL, 4.00 eq) in DCM (50.0 mL). The mixture was agitated with N_2_ for 2 h at rt. Then, MeOH (7.00 mL) was added and agitated with N_2_for another 30 min. The resin was washed with DMF (50.0 mL x 3). Then 20% piperidine in DMF (50.0 mL) was added and the mixture was agitated with N_2_ for 20 min at 20°C. Then the mixture was filtered. The resin was washed with DMF (50.0 mL x 3) to remove residual piperidine.

**Coupling**: A solution of Fmoc-Ser(OtBu)-OH (7.80 g, 20.4 mmol, 3.00 eq) and HBTU (6.50 g, 19.4 mmol, 2.85 eq) in DMF (50 mL) was added DIPEA (40.8 mmol, 7.00 mL, 6.00 eq) to the resin and agitated with N2 for 30 min at rt. The resin was then washed with DMF (50.0 mL x 4). The coupling and deprotection steps were repeated with Fmoc-Ala-OH and 3-hydroxymyristic acid. The resin was washed with MeOH (50.0 mL x 3) and dried under vacuum. Then 50.0 mL of cleavage buffer (20% HFIP/ 80% DCM) was added to the flask containing the side chain protected peptide resin at rt. The cleavage was performed twice. The crude peptide was concentrated under pressure to give the compound **1** (2.30 g, crude) as a white solid and further purified by RP-HPLC.

^1^H NMR (600 MHz, Methanol-*d4*) δ 4.45 (dt, *J* = 19.8, 5.3 Hz, 1H), 4.42 – 4.35 (m, 2H), 3.98 (dqd, *J* = 10.0, 5.2, 2.4 Hz, 1H), 3.68 (ddd, *J* = 11.6, 9.1, 5.1 Hz, 1H), 3.60 (ddd, *J* = 9.0, 5.5, 3.1 Hz, 1H), 2.43 – 2.28 (m, 2H), 1.47 (h, *J* = 10.6, 9.7 Hz, 3H), 1.41 (dd, *J* = 7.2, 4.1 Hz, 3H), 1.38 (dd, *J* = 7.2, 3.3 Hz, 3H), 1.30 (s, 17H), 1.20 (d, *J* = 1.7 Hz, 9H), 0.90 (t, *J* = 7.0 Hz, 3H). HRMS (*m/z*): [M]^-^ calcd. for C_27_H_51_N_3_O_7_, 528.3654; found, 528.3653.

Methyl 2-(4-((*tert*-butoxycarbonyl)amino)phenyl)thiazole-4-carboxylatebenzoate (**3**)

To a solution of compound **2** (7.69 g, 32.4 mmol, 1.20 eq), compound **2-1** (6 g, 27.0 mmol, 1.00 eq) and K_3_PO_4_ (1.5 M, 54.o mL, 3.00 eq) in THF (120 mL) was added SPhos Pd G3 (2.11 g, 2.70 mmol, 0.1 eq). The reaction was stirred at 60 °C for 14 h under nitrogen. The reaction was monitored by TLC analysis (1:3 EtOAc/petroleum spirit) following disappearance of starting material **2** and appearance of product compound **3** (R_f_ 0.30). The reaction mixture was poured into water (200 mL) and extracted with EtOAc (3 x 100 mL). The combined organic phase was washed with brine (150 mL), dried over Na_2_SO_4_, filtered, and concentrated. The residue was purified by flash silica chromatography to yield compound **3** (5.40 g, 16.1 mmol, 59.77%) as a white solid.

^1^H NMR (600 MHz, DMSO-*d_6_*) δ 8.50 (s, 1H), 7.87 (d, *J* = 8.8 Hz, 2H), 7.61 (d, *J* = 8.4 Hz, 2H), 3.86 (s, 3H), 1.49 (s, 9H). HRMS (*m/z*): [M]^-^ calcd. for C_16_H_18_N_2_O_4_S, 333.0915; found, 333.0909.

*tert*-butyl (4-(4-carbamoylthiazol-2-yl)phenyl)carbamate (**4**)

To a solution of compound **S13** (5.40 g, 16.15 mmol, 1.00 eq) in MeOH (100 mL) was added 30-33% NH_4_OH (45.5 g, 1.30 mol, 50 mL, 80.4 eq), and the reaction was stirred at 50 °C for 12 h. The reaction was monitored by LCMS following disappearance of starting material **3** and appearance of product compound **4** (Rt = 0.874 min, m/z = 320 (M+1)^+^). The reaction mixture was concentrated to yield compound **4** (5.1 g, 15.9 mmol, 98.8% yield) as a white solid.

^1^H NMR (600 MHz, DMSO-*d_6_*) δ 8.19 (s, 1H), 7.93 (d, *J* = 8.7 Hz, 2H), 7.60 (d, *J* = 8.6 Hz, 2H), 1.49 (s, 9H). HRMS (*m/z*): [M]^-^ calcd. for C_15_H_17_N_3_O_3_S, 318.0918; found, 318.0922.

*tert*-butyl (4-(4-carbamothioylthiazol-2-yl)phenyl)carbamate (**5**)

To a solution of compound **4** (5.00 g, 15.6 mmol, 1.00 eq) in THF (100 mL) was added Lawesson’s reagent (3.80 g, 9.39 mmol, 0.6 eq) and stirred at 60 °C for 14 h under N_2_. The reaction was monitored by LCMS following disappearance of starting material **4** and appearance of product compound **5** (Rt = 0.966 min, *m/z* = 336 (M+1)^+^). The reaction mixture was concentrated, and the residue purified by trituration in DCM (50 mL) for 2 h at rt. The triturated product was filtered, and the filter-cake was collected yielding compound **5** (3.30 g, 9.84 mmol, 62.8% yield) as a yellow solid.

^1^H NMR (600 MHz, DMSO-*d_6_*) δ 10.03 (s, 1H), 9.67 (s, 1H), 9.58 (s, 1H), 8.39 (s, 1H), 8.08 – 7.88 (m, 2H), 7.60 (d, *J* = 8.5 Hz, 2H), 1.49 (s, 9H). HRMS (*m/z*): [M]^-^ calcd. for C_15_H_17_N_3_O_2_S_2_, 334.0689; found, 334.0697.

ethyl 2'-(4-((tert-butoxycarbonyl)amino)phenyl)-[2,4'-bithiazole]-4-carboxylate (**6**)

To a solution of compound **5** (2.70 g, 8.05 mmol, 1.00 eq) dissolved in DME (190 mL) under N_2_, was added KHCO_3_(6.45 g, 64.4 mmol, 8.00 eq) and stirred for 5 min. Then, ethyl-3-bromo-oxopropanoate (4.71 g, 24.1 mmol, 3.02 mL, 3.00 eq) was dissolved in additional DME (50 mL) and added dropwise (approx. 1mL/min) to the reaction. The reaction mixture was stirred for 16 h at rt. The desired hydroxythiazoline intermediate was concentrated in vacuo, re-dissolved in EtOAc, and extracted with brine. The combined organic phases were dried over Na_2_SO_4_, filtered and concentrated in vacuo. The crude hydroxythiazoline intermediate was dissolved in DME (190 mL) and stirred at 0 °C for 10 min. Then, pyridine (5.73 g, 72.4 mmol, 5.85 mL, 9 eq) was added to reaction mixture (0.1 mL/min) and stirred at 0 °C for an additional 15 min. TFAA (6.76 g, 32.2 mmol, 4.48 mL, 4.00 eq) was added to the reaction mixture (0.1 mL/min) and stirred at 0 °C for an additional 2 h. Finally, TEA (1.63 g, 16.1 mmol, 2.24 mL, 2.00 eq) was added to the reaction drop-wise (0.1 mL/min) and run at 0 °C to 20 °C for 12 h. The reaction was monitored by LCMS following disappearance of starting material **5** and appearance of product compound **6** (Rt = 1.043 min, *m/z* = 432 (M+1)^+^). The reaction mixture was concentrated, the residue was re-dissolved in EtOAc (200 mL), washed with 1 M HCl (100 mL), saturated NaHCO_3_ (100 mL), brine (100 mL), dried with Na_2_SO_4_, filtrated and concentrated. The residue was purified by trituration with MTBE (50 mL) for 1 h under at rt. The triturated product was filtered, and the filter-cake collected yielding compound **6** (2.90 g, 6.72 mmol, 83.5% yield) as a brown solid.

^1^H NMR (600 MHz, DMSO-*d_6_*) δ 9.70 (s, 1H), 8.57 (s, 1H), 8.34 (s, 1H), 7.91 (d, *J* = 8.4 Hz, 2H), 7.63 (d, *J* = 8.4 Hz, 2H), 4.34 (q, *J* = 7.1 Hz, 2H), 1.49 (s, 9H), 1.33 (t, *J* = 7.1 Hz, 3H). HRMS (*m/z*): [M]^-^ calcd. for C_20_H_21_N_3_O_4_S_2_, 430.0901; found, 430.0922.

ethyl 2'-(4-aminophenyl)-[2,4'-bithiazole]-4-carboxylate (**7**)

To a solution of compound **6** (400 mg, 927 µmol, 1.00 eq) in DCM (1.00 mL) was added TFA (3.70 g, 32.4 mmol, 2.40 mL, 34.97 eq), the reaction mixture was stirred at rt for 1 hr. The reaction was monitored by TLC (1:3 EtOAc/petroleum spirit) following the disappearance of starting compound **6** (Rf 0.3) and appearance of product compound **7** as one spot (Rf 0.15). The reaction mixture was concentrated *in vacuo* to yield compound **7** (400 mg, 898 µmol, 96.9% yield, TFA) as a light yellow solid.

^1^H NMR (600 MHz, MeOD-*d_4_*) δ 8.39 (s, 1H), 8.18 (s, 1H), 7.85 (d, *J* = 8.1 Hz, 2H), 6.92 (d, *J* = 7.9 Hz, 2H), 4.42 (q, *J* = 7.1 Hz, 2H), 1.42 (t, *J* = 7.2 Hz, 3H). HRMS (*m/z*): [M]^-^ calcd. for C_15_H_13_N_3_O_2_S_2_, 330.0376; found, 330.0390.

2-hydroxy-3-isopropoxy-4-nitrobenzaldehyde (**9**)

A solution of compound **8** (10 g, 45.0 mmol, 1.00 eq) in DCM (50.0 mL) was added dropwise to fuming nitric acid at -40 °C and stirred for 4 h. The reaction was monitored by TLC analysis (1:3 EtOAc/petroleum spirit), following disappearance of starting material **8** (R_f_ 0.55), and appearance of product compound **9** (R_f_ 0.50). The reaction was quenched with ice-water (50.0 mL) and diluted with DCM. The phases were separated, and the aqueous phase was extracted with DCM (3 x 100 mL). The combined organic phases were concentrated *in vacuo* to a brown residue. The residue was purified by flash silica chromatography to yield compound **9** (8.00 g, 35.5 mmol, 79.0%) as a light-yellow oil.

^1^H NMR (400 MHz, CDCl_3_) δ 11.43 (s, 1H), 9.97 (s, 1H), 7.39 (d, *J* = 8.4 Hz, 1H), 7.24 (t, *J* = 8.2 Hz, 1H), 4.88 (hept, *J* = 6.2 Hz, 1H), 1.32 (d, *J* = 6.2 Hz, 6H).

2-(allyloxy)-3-isopropoxy-4-nitrobenzaldehyde (**10**)

To a solution of compound **9** (8.00 g, 35.5 mmol, 1.00 eq) in DMF (50.0 mL) was added K_2_CO_3_ (9.82 g , 71.1 mmol, 2.00 eq) and 3-bromoprop-1-ene (5.16 g, 42.6 mmol, 1.20 eq). The reaction was stirred for 2 h at rt. The reaction was monitored by TLC (1:3 EtOAc/petroleum spirit), following disappearance of starting material **9 (**R_f_ 0.55) and appearance of product compound **10** (R_f_ 0.70). After completion, the reaction was poured over H_2_O (200 mL), and the phases separated. The aqueous phase was extracted with EtOAc (3x150 mL) and the combined organic phases were washed with brine (3 x 100 mL), dried over Na_2_SO_4_, and concentrated *in vacuo.* The residue was purified by flash silica chromatography to yield compound **10** (6.10 g, 23.0 mmol, 64.7%) as a light yellow oil.

^1^H NMR (400 MHz, CDCl_3_) δ 10.39 (d, *J* = 0.9 Hz, 1H), 7.63 (d, *J* = 8.5 Hz, 1H), 7.50 (dd, *J* = 8.6, 0.9 Hz, 1H), 6.05 (ddt, *J* = 16.6, 10.3, 6.1 Hz, 1H), 5.48 – 5.23 (m, 2H), 4.73 (dt, *J* = 6.1, 1.2 Hz, 2H), 4.68 (hept, 1H), 1.32 (d, *J* = 6.2 Hz, 6H).

2-(allyloxy)-3-isopropoxy-4-nitrobenzoic acid (**11**)

A solution of compound **10** (6.10 g, 23.0 mmol, 1.00 eq) and 2-methylbut-2-ene (17.7 g, 253 mmol, 26.8 mL, 11.0 eq) in tButOH (150 mL) was prepared at rt. Then, a solution of sodium chlorite (2.94 g, 27.6 mmol, 85.0% purity, 1.2 eq) in aqueous NaH_2_PO_4_ (1.00 M, 26.8 mL, 1.17 eq) was added dropwise. The reaction was stirred for 1 h at rt. The reaction was monitored by TLC ((1:3 EtOAc/petroleum spirit) following disappearance of starting material **10** and appearance of the product compound **11** as a single major peak (R_f_ 0.01). The reaction mixture was quenched by addition of aqueous Na_2_SO_3_ (14.0 mmol in 500 mL). The mixture was diluted with EtOAc (100 mL) and the pH adjusted to 4 with 1N HCl. The aqueous phase was extracted with EtOAc (50 mL) and the combined organic phases were washed with brine (150 mL) dried over Na_2_SO_4_, filtered, and concentrated *in vacuo* to provide compound **11** (6.00 g, 21.3 mmol, 92.8%) as a brown oil.

^1^H NMR (400 MHz, CDCl_3_) δ 7.89 (d, *J* = 8.7 Hz, 1H), 7.56 (d, *J* = 8.7 Hz, 1H), 6.08 (ddt, *J* = 16.7, 10.2, 6.3 Hz, 1H), 5.52 – 5.34 (m, 2H), 4.80 (dt, *J* = 6.4, 1.1 Hz, 2H), 4.64 (hept, *J* = 12.4, 6.1 Hz, 1H), 1.32 (d, *J* = 6.1 Hz, 6H).

Allyl 4-aminobenzoate (**11-2**)

To a solution of compound **S11-1** (25.0 g, 182 mmol, 1.00 eq) suspended in DMF (125 mL) was added K_2_CO_3_ (75.6 g, 547 mmol, 3.00 eq) and 3-bromoprop-1-ene (24.3 g, 201 mmol, 1.10 eq). The reaction mixture was stirred at 80 °C for 2 h. The reaction was monitored by TLC analysis (1:3 EtOAc/petroleum spirit) following the disappearance of starting material **S11-1** (R_f_ 0.01) and appearance of two spots (R_f_ 0.4, R_f_ 0.55). The reaction was poured onto H_2_O (500 mL), and the aqueous phase extracted with EtOAc (3 x 150 mL). The combined organic phases were washed with brine (3 x 100 mL), dried over Na_2_SO_4_, and concentrated *in vacuo* to a yellow solid. The residue was purified by flash silica chromatography to yield compound **11-2** (22.0 g, 124 mmol, 68.1%) as a light yellow solid.

^1^H NMR (400 MHz, CDCl_3_) *δ*: 7.90-7.88 (d, *J* = 8 Hz, 2H), 6.66-6.64 (d, *J* = 8 Hz, 2H), 6.10-6.00 (m, 1H), 5.42-5.38 (d, *J* = 16 Hz, 1H), 5.28-5.25 (d, *J* = 12 Hz, 1H), 4.79-4.77 (d, *J* = 8 Hz, 2H), 4.07 (s, 2H).

Allyl 4-(2-(allyloxy)-3-isopropoxy-4-nitrobenzamido)benzoate (**12**)

To a solution of compound **11** (5.40 g, 19.2 mmol, 1.00 eq) in DCM (60.0 mL) was added DIPEA (4.96 g, 38.4 mmol, 2.00 eq) and POCl_3_ at 0 °C for 0.5 h. Then, compound **S11-2** (3.40 g, 19.2 mmol, 1.00 eq) in DCM (30.0 mL) was added dropwise to the solution. The reaction was stirred for 1 h at 0 °C. The reaction was monitored by TLC analysis (1:3 EtOAc/petroleum spirit) following the disappearance of starting material **11** (R_f_ 0.01) and appearance of product compound **12** (R_f_ 0.5) as a one main spot. The reaction was quenched with the addition of saturated NaHCO_3_ (100 mL) at a dropwise rate. The phases were separated, and the organic phase was washed with NaHCO_3_ (1x100 mL), brine (1x100 mL), dried over Na_2_SO_4_, and concentrated *in vacuo*. The residue was purified by flash silica chromatography to yield compound **12** (5.00 g , 11.4 mmol, 59.1%) as a light yellow solid.

^1^H NMR (400 MHz, CDCl_3_) δ 10.16 (s, 1H), 8.08 (dd, *J* = 8.8, 1.3 Hz, 3H), 7.75 (d, *J* = 8.8 Hz, 2H), 7.63 (d, *J* = 8.8 Hz, 1H), 6.20 – 6.07 (m, 1H), 6.07 – 5.94 (m, 1H), 5.50 (dq, *J* = 17.1, 1.4 Hz, 1H), 5.46 – 5.38 (m, 2H), 5.30 (dq, *J* = 10.5, 1.3 Hz, 1H), 4.82 (dt, *J* = 5.7, 1.5 Hz, 2H), 4.79 (dt, *J* = 6.1, 1.2 Hz, 2H), 4.65 (hept, *J* = 6.2 Hz, 1H), 1.35 (d, *J* = 6.2 Hz, 6H).

Allyl 4-(2-(allyloxy)-4-amino-3-isopropoxybenzamido)benzoate (**13**)

To a solution of compound **12** (2.50 g, 5.68 mmol, 1.00 eq) in EtOAc (100 mL) was added stannous chloride (6.40 g, 28.4 mmol, 5.00 eq). The reaction mixture was stirred at 60 °C for 2 h. The reaction was monitored by TLC analysis (1:5 EtOAc/petroleum spirit) following the disappearance of starting material **12** (R_f_ 0.5) and appearance of two spots (R_f_ 0.05, R_f_ 0.01 ). The reaction was diluted with saturated NaHCO_3_ (100 mL), and the phases separated. The aqueous layer was extracted with EtOAc (3 x 100 mL) and the combined organics were washed with brine (1 x 100 mL), dried over Na_2_SO_4,_ filtered and concentrated *in vacuo*. The residue was purified by flash silica chromatography to yield compound **13** (1.5 g, 3.65 mmol, 64.4%) as a light yellow oil.

^1^H NMR (400 MHz, CDCl_3_) δ 10.27 (s, 1H), 8.04 (dt, *J* = 5.1, 2.3, 2.3 Hz, 2H), 7.84 (d, *J* = 8.7 Hz, 1H), 7.74 (dt, *J* = 5.0, 2.3, 2.2 Hz, 2H), 6.62 (d, *J*= 8.7 Hz, 1H), 6.21 – 6.10 (m, 1H), 6.10 – 5.98 (m, 1H), 5.49 (dq, *J* = 17.2, 1.5 Hz, 1H), 5.45 – 5.35 (m, 2H), 5.28 (dq, *J* = 10.4, 1.3 Hz, 1H), 4.81 (dt, *J* = 5.6, 1.4 Hz, 2H), 4.69 (dt, *J* = 5.8, 1.3 Hz, 2H), 4.58 (hept, *J* = 6.3 Hz, 1H), 1.34 (d, *J* = 6.2 Hz, 6H). HRMS (*m/z*): [M]^-^ calcd. for C_23_H_26_N_2_O_5_, 409.1769; found, 409.1745.

Allyl 4-(2-(allyloxy)-3-isopropoxy-4-(4-nitrobenzamido)benzamido)benzoate (**S7**)

To a solution of *p*-nitrobenzoic acid (1.46 g, 8.76 mmol, 1.2 eq) in DCM (15.0 mL) was added SOCl_2_ (4.34 g, 36.5 mmol, 2.65 mL, 5.00 eq) and one drop of DMF. The reaction mixture was stirred at for 0.5 h at rt. The reaction mixture was then concentrated to give a residue of the acid chloride. The residue was suspended in DCM (15.0 mL), then DIPEA (2.83 g, 21.9 mmol, 3.82 mL, 3.00 eq) was added to the reaction mixture followed by compound **13** and stirred for 0.5 h at RT. The reaction was monitored by LCMS following disappearance of starting material **13** appearance of product compound **14** as a single peak. TLC analysis (1:3 EtOAc/petroleum spirit) following the disappearance of starting material (R_f_ 0.01) an appearance of two spots (R_f_ 0.40, R_f_ 0.05). The reaction mixture was adjusted to a pH = 3 with 1N HCl. The mixture was filtered, and the filtrate was extracted with EtOAc (3 x 50mL), and the pooled organics were concentrated *in vacuo.* The residue was purified by flash silica chromatography to yield compound **14** (4.00 g, 7.15 mmol, 97.8%) as a light yellow solid.

^1^H NMR (400 MHz, DMSO-*d_6_*) δ 10.61 (s, 1H), 10.15 (s, 1H), 8.39 (d, *J* = 8.8 Hz, 2H), 8.21 (d, *J* = 8.9 Hz, 2H), 7.99 (d, *J* = 8.8 Hz, 2H), 7.87 (d, *J* = 8.8 Hz, 2H), 7.69 (d, *J* = 8.4 Hz, 1H), 7.41 (d, *J* = 8.4 Hz, 1H), 6.03 (dddt, *J* = 17.2, 14.0, 10.7, 5.5 Hz, 2H), 5.38 (dp, *J* = 15.7, 1.7, 1.4, 0.1 Hz, 2H), 5.27 (ddt, *J* = 10.5, 3.9, 1.9 Hz, 1H), 5.19 (dd, *J* = 10.5, 1.5 Hz, 1H), 4.79 (dt, *J* = 5.5, 1.5 Hz, 2H), 4.61 (dd, *J* = 5.7, 1.7 Hz, 2H), 4.48 (m, 1H), 1.24 (d, *J* = 6.2 Hz, 6H). ESI-MS [M + H]^+^: *m/z =* 560.

Allyl 4-(2-(allyloxy)-4-(4-aminobenzamido)-3-isopropoxybenzamido)benzoate (**15**)

To a solution of compound **14** (4.00 g, 7.15 mmol, 1.00 eq) in EtOAc (160mL) was added SnCl_2_ dihydrate (8.07 g, 35.7 mmol, 5.00 eq), the reaction mixture was stirred for 1 h at 60°C. The reaction was monitored by TLC analysis (1:3 EtOAc/petroleum spirit) following the disappearance of starting material **14** (R_f_ 0.4) and appearance of product compound **15** (R_f_ 0.10). The reaction was diluted with saturated NaHCO­_3_ (100 mL), and the phases separated. The aqueous layer was extracted with EtOAc (3 x 100 mL) and the combined organics were washed with brine (1 x 100 mL), dried over Na_2_SO_4_, filtered and concentrated *in vacuo*. The crude product was triturated with EtOAc/petroleum spirit (1:10, 30.0 mL) for 1 h at 25 °C to give compound **15** (3.20 g, 6.04 mmol, 84.5% yield) as a light yellow solid.

^1^H NMR (400 MHz, DMSO-*d_6_*) δ 10.54 (s, 1H), 9.06 (s, 1H), 7.97 (dd, *J* = 12.2, 8.5 Hz, 3H), 7.87 (d, *J* = 8.5 Hz, 2H), 7.70 (d, *J* = 8.3 Hz, 2H), 7.40 (d, *J* = 8.5 Hz, 1H), 6.63 (d, *J* = 8.2 Hz, 2H), 6.04 (dp, *J* = 16.7, 5.4 Hz, 2H), 5.88 (s, 2H), 5.39 (dd, 2H), 5.24 (dd, *J* = 28.5, 10.4 Hz, 2H), 4.79 (d, *J* = 5.3 Hz, 2H), 4.60 (d, *J* = 5.5 Hz, 2H), 4.54 (hept, *J* = 6.1 Hz, 1H), 1.28 (d, *J* = 6.1 Hz, 6H). ESI-MS [M + H]^+^: *m/z =* 530.

Allyl (*S*)-4-(2-(allyloxy)-4-(4-(2-((*tert*-butoxycarbonyl)amino)-4-oxo-4-(tritylamino)butanamido)benzamido)-3-isopropoxybenzamido)benzoate (**S15-1**)

To a solution of *N*^2^-(*tert*-butoxycarbonyl)-*N*^4^-trityl-*L*-asparagine and compound **15** in DCM (35.0 mL) was added propylphosphonic anhydride (4.24 g , 6.67 mmol, 3.96 mL , 50% purity, 1.00 eq) and pyridine (1.58 g, 20.0 mmol, 1.61 mL, 3.00 eq). The reaction mixture was stirred for 12 h at 40°C. The reaction was monitored by LCMS following disappearance of starting material **15** appearance of product compound **S15-1** as a single peak (Rt = 1.203 min, *m/z* = 986 (M+1)^+^). The reaction mixture was extracted with DCM (3 x 50.0 mL) and the pooled organics were concentrated *in vacuo* to give a residue. The residue was purified by flash silica chromatography (1:5 – 1:3 EtOAc/Petroleum spirit) to yield compound **S15-1** (4.00 g, 7.15 mmol, 97.8%) as a light yellow solid.

^1^H NMR (600 MHz, MeOD-*d_4_*) δ 8.08 (d, *J* = 8.7 Hz, 1H), 8.03 (d, *J* = 8.7 Hz, 2H), 7.93 (d, *J* = 8.5 Hz, 2H), 7.82 (d, *J* = 8.5 Hz, 2H), 7.76 (d, *J* = 8.5 Hz, 2H), 7.69 (d, *J* = 8.6 Hz, 1H), 7.23 (d, *J* = 4.3 Hz, 15H), 6.10 (dddd, *J* = 22.8, 21.6, 10.6, 5.7 Hz, 2H), 5.43 (td, *J* = 18.6, 17.0, 1.6 Hz, 2H), 5.32 – 5.26 (m, 2H), 4.81 (dt, *J* = 5.6, 1.5 Hz, 2H), 4.72 (d, 3H), 4.55 (t, *J* = 6.9 Hz, 1H), 2.82 (d, *J* = 6.9 Hz, 2H), 1.46 (s, 9H), 1.35 (d, *J* = 6.2 Hz, 6H). HRMS (*m/z*): [M]^-^ calcd. for C_58_H_59_N_5_O_10_, 985.4189; found, 985.4192.

Allyl (*S*)-4-(2-(allyloxy)-4-(4-(2,4-diamino-4-oxobutanamido)benzamido)-3-isopropoxybenzamido)benzoate (**16**)

To a solution of compound **S15-1** (2.50 g, 2.54 mmol, 1.00 eq) dissolved in DCM (13.0 mL) was added TFA (13.0 mL), the reaction mixture was stirred for 12 h at rt. The reaction was monitored by TLC analysis (1:3 EtOAc/petroleum spirit) following disappearance of starting material **S15-1** and appearance of product compound **16** as a single peak (R_f_ 0.20). The reaction mixture was concentrated *in vacuo* to give a residue which was purified by prep-HPLC to give compound **16** (1.40 g, 1.85 mmol, 72.8% yield, TFA salt) as white solid.

^1^H NMR (600 MHz, MeOD-*d_4_*) δ 8.06 (d, *J* = 8.7 Hz, 1H), 8.03 (d, *J* = 8.7 Hz, 2H), 7.97 (d, *J* = 8.7 Hz, 2H), 7.82 (d, *J* = 8.7, 1.7 Hz, 4H), 7.69 (d, *J* = 8.7 Hz, 1H), 6.17 – 5.97 (m, 2H), 5.43 (td, *J* = 17.3, 15.8, 1.6 Hz, 2H), 5.29 (ddd, *J* = 10.5, 5.6, 1.4 Hz, 2H), 4.81 (d, *J* = 5.6 Hz, 2H), 4.72 (d, 3H), 4.38 (dd, *J* = 9.0, 4.5 Hz, 1H), 3.03 (dd, *J* = 17.1, 4.5 Hz, 1H), 2.89 (dd, *J* = 17.0, 9.0 Hz, 1H), 1.35 (d, *J* = 6.2 Hz, 6H). HRMS (*m/z*): [M]^-^ calcd. for C_34_H_37_N_5_O_8_, 642.2569; found, 642.2551.

Ethyl 2'-(4-((2S)-2-((2S)-3-(tert-butoxy)-2-((2S)-2-(3-hydroxytetradecanamido)propanamido)propanamido)propanamido)phenyl)-[2,4'-bithiazole]-4-carboxylate (**S17**)

To a solution of compound **1** (1.75 g, 2.47 mmol, 74.6% purity, 1.00 eq) in THF (20.0 mL) was added TEA (279 mg, 2.77 mmol, 384 µL, 1.12 eq) and Isobutyl chloroformate (IBCF) (349 mg, 2.56 mmol, 335 µL, 1.04 eq) at 0 °C for 30 min. Then, compound **7** (1.40 g, 3.14 mmol, 1.27 eq, TFA) in THF (20.0 mL) was added to the solution, and the reaction mixture was stirred at 0 °C for 1 h. The reaction was monitored by LCMS following disappearance of starting compound **1** and appearance of product compound **S17** MS (Rt = 1.170 min, *m/z* = 843 (M+1)+) was detected. The reaction mixture was concentrated *in vacuo* to give a residue. The residue was purified by prep-HPLC to give the compound **S17** (1.10 g, 1.17 mmol, 47.5% yield, 89.8% purity) as a brown solid and was used directly for next reaction.

Ethyl 2'-(4-((2S)-2-((2S)-3-(tert-butoxy)-2-((2S)-2-(3-hydroxytetradecanamido)propanamido)propanamido)propanamido)phenyl)-[2,4'-bithiazole]-4-carboxylate (**18**)

To a solution of compound **S17** (1.10 g, 1.17 mmol, 89.8% purity, 1.00 eq) in THF (10.0 mL) and H2O (10.0 mL) was added LiOH.H2O (150 mg, 3.58 mmol, 3.05 eq) and the reaction mixture was stirred at rt for 1 h. The reaction was monitored by LCMS following the disappearance of reactant compound **S17** and appearance of the product compound **S18** MS (Rt = 1.018 min, *m/z* = 815 (M+1)+) was detected. The reaction mixture was adjusted to pH = 3 with 1N HCl and extracted with EtOAc (50.0 mL x 3). The organic layer was concentrated *in vacuo* to give compound **S18** (950 mg, 1.13 mmol, 96.5% yield, 97.1% purity) as a brown solid.

^1^H NMR (600 MHz, DMSO-*d_6_*) δ 9.97 (s, 1H), 8.51 (s, 1H), 8.32 (s, 1H), 7.95 (d, *J* = 8.7 Hz, 2H), 7.65 (d, *J* = 8.5 Hz, 2H), 5.75 (s, 2H), 4.36 – 4.26 (m, 1H), 4.26 – 4.15 (m, 1H), 4.03 (q, *J* = 7.1 Hz, 1H), 3.91 (d, *J* = 6.6 Hz, 2H), 3.86 – 3.72 (m, 2H), 2.19 (h, *J* = 7.4, 6.3 Hz, 2H), 1.99 (s, 1H), 1.99 – 1.86 (m, 1H), 1.27 -1.18 (m, 21H), 1.13 – 1.04 (m, 7H), 0.95 (d, *J* = 6.7 Hz, 6H), 0.85 (t, *J* = 6.9 Hz, 3H).

Allyl 4-(2-(allyloxy)-4-(4-((2S)-4-amino-2-(2'-(4-((2S)-2-((2S)-3-(tert-butoxy)-2-((2S)-2-(3-hydroxytetradecanamido)propanamido)propanamido)propanamido)phenyl)-[2,4'-bithiazole]-4-carboxamido)-4-oxobutanamido)benzamido)-3-isopropoxybenzamido)benzoate (**S19**)

To a solution of compound **S18** (930 mg, 1.11 mmol, 97.1% purity, 1.00 eq) and compound **16** (1.35 g, 1.78 mmol, 1.61 eq, TFA) in DMF (20.0 mL) was added DIEA (434 mg, 3.36 mmol, 584 uL, 3.03 eq) and HBTU (421 mg, 1.11 mmol, 1.00 eq), the reaction mixture was stirred at rt for 0.5 h. The reaction was monitored by LCMS following the disappearance of compound **S18** and the appearance of compound **S19** MS (Rt = 3.025 min, *m/z* = 1440 (M+1)^+^) was detected. The reaction mixture was concentrated *in vacuo* to give a residue. The residue was purified by prep-HPLC to give the compound **S19** (350 mg, 242.93 µmol, 17.0% yield) was obtained as a white solid.

^1^H NMR (400 MHz, DMSO-*d_6_*) δ 10.58 (s, 1H), 10.47 (s, 1H), 10.12 (d, *J* = 5.7 Hz, 1H), 9.53 (s, 1H), 8.68 (d, *J* = 7.8 Hz, 1H), 8.39 (s, 1H), 8.33 (s, 1H), 8.15 (dd, *J* = 25.0, 7.0 Hz, 1H), 8.01 – 7.95 (m, 4H), 7.87 (d, *J* = 8.9 Hz, 2H), 7.81 (dd, *J* = 8.7, 1.8 Hz, 5H), 7.55 – 7.48 (m, 1H), 7.40 (d, *J* = 8.5 Hz, 1H), 7.03 – 6.98 (m, 1H), 6.87 (s, 1H), 6.63 (s, 1H), 6.04 (m, 2H), 5.39 (td, *J* = 17.0, 15.1, 1.7 Hz, 2H), 5.28 (dt, *J*= 10.5, 1.5 Hz, 1H), 5.20 (dd, *J* = 10.4, 1.6 Hz, 1H), 4.96 (q, *J* = 6.9 Hz, 1H), 4.79 (dt, *J* = 5.4, 1.6 Hz, 2H), 4.67 – 4.56 (m, 2H), 4.48 (hept, *J* = 13.0, 6.4 Hz, 2H), 4.32 (q, *J* = 7.2 Hz, 2H), 3.79 (s, 1H), 3.56 – 3.40 (m, 2H), 2.87 – 2.65 (m, 2H), 2.22 (t, *J* = 5.8 Hz, 2H), 2.18 (s, 2H), 1.35 (s, 17H), 1.29 – 1.19 (m, 20H), 1.11 (d, *J* = 1.2 Hz, 7H), 0.84 (t, *J* = 6.7 Hz, 3H).

4-(4-(4-((2*S*)-4-amino-2-(2'-(4-((2*S*)-2-((2*S*)-3-(*tert*-butoxy)-2-((2*S*)-2-(3-hydroxytetradecanamido)propanamido)propanamido)propanamido)phenyl)-[2,4'-bithiazole]-4-carboxamido)-4-oxobutanamido)benzamido)-2-hydroxy-3-isopropoxybenzamido)benzoic acid (**20**)

To a solution of compound **S19** (340 mg, 234 umol, 99.4% purity, 1.00 eq) in DMF (1.00 mL) was added morpholine (136 mg, 1.56 mmol, 137, 6.65 eq) and palladium triphenylphosphine (102 mg, 88.2 umol, 3.76e-1 eq). The reaction mixture was stirred at rt for 1 h in the dark. The reaction was monitored by LCMS following the disappearance of compound **S19** and the appearance of product compound **S20** (Rt = 0.958 min, *m/z* = 1360 (M+1)^+^). The reaction mixture was concentrated *in vacuo* to give a residue. The residue was purified by prep-HPLC to give the compound **S20** (190 mg, 108 µmol, 46.4% yield, 78.0% purity) as a white solid and was used directly for the next reaction.

4-(4-(4-((2*S*)-4-amino-2-(2'-(4-((2*S*)-2-((2*S*)-3-hydroxy-2-((2*S*)-2-(3-hydroxytetradecanamido)propanamido)propanamido)propanamido)phenyl)-[2,4'-bithiazole]-4-carboxamido)-4-oxobutanamido)benzamido)-2-hydroxy-3-isopropoxybenzamido)benzoic acid (**Lapcin**)

To a solution of compound **S20** (50.0 mg, 28.6 umol, 78.0% purity, 1.00 eq) in DCM (0.90 mL) was added TFA (0.30 mL), and the reaction mixture was stirred at rt for 0.5 hr. The reaction was monitored by LCMS following the disappearance of reactant compound **S20** and appearance of final product compound **lapcin** MS (Rt = 0.825 min, *m/z* = 1305 (M+1)^+^). The reaction mixture was concentrated *in vacuo* to yield a residue. The residue was purified by prep-HPLC to give the compound **Lapcin**, (10.0 mg, 4.91 µmol, 17.0% yield) as a white solid.

^1^H NMR (600 MHz, DMSO-*d_6_*) δ δ 12.79 (sb, 1H), 12.32 (s, 1H), 10.69 (s, 1H), 10.51 (s, 1H), 10.08 – 9.82 (m, 1H), 9.43 (s, 1H), 8.70 (s, 2H), 8.39 (dt, *J* = 36.0, 9.6 Hz, 3H), 8.17 (ddd, *J* = 28.5, 15.7, 6.3 Hz, 4H), 8.00 (dq, *J* = 13.9, 7.0 Hz, 8H), 7.86 (dt, *J* = 21.8, 7.2 Hz, 7H), 7.79 – 7.63 (m , 2H), 7.56 (s, 2H), 7.37 – 7.16 (m, 2H), 7.05 (s, 1H), 5.27 – 5.07 (m, 1H), 5.08 – 4.92 (m, 2H), 4.70 – 4.49 (m, 2H), 4.44 (m, *J* = 6.8 Hz, 2H), 4.40 – 4.19 (m, 3H), 3.90 – 3.76 (m, 2H), 3.76 – 3.58 (m, 4H), 3.03 – 2.68 (m, 5H), 2.39 – 1.82 (m, 3H), 1.51 – 1.04 (m, 44H), 0.85 (hept, *J* = 7.4 Hz, 5H).

^13^C NMR (400 MHz, DMSO-*d_6_*) δ ^13^C NMR (151 MHz, DMSO) δ 172.9, 171.8, 171.4, 171.3, 170.1, 169.9, 168.5, 168.2, 166.9, 164.2, 162.3, 162.0, 160.2, 157.9, 154.1, 150.2, 148.3, 142.4, 142.0, 141.1, 137.0, 136.3, 130.2, 128.4, 127.2, 126.3, 125.1, 122.8, 120.7, 119.6, 119.0, 117.7, 112.4, 112.2, 74.9, 67.8, 67.4, 61.5, 55.3, 55.2, 51.0, 49.3, 48.7, 43.5, 40.3, 40.0, 39.9, 39.8, 39.7, 39.5, 39.4, 39.2, 39.1, 36.8,9 31.3, 29.1, 29.0, 28.7, 25.2, 25.0, 22.3, 22.1, 17.8, 13.9.

HRMS (*m/z*): [M]^-^ calcd. for C_65_H_79_N_11_O_15_S_2_, 1302.4969; found, 1302.4957.

**Supplementary Table 1: Lapcin biosynthetic gene cluster gene annotation**

| ORF | Gene size (bp) | Gene name | Proposed function | Protein, [Source Organism], Accession number | ID % |
| --- | --- | --- | --- | --- | --- |
| 1 | 837 | Lap A | Self-resistance protein | pentapeptide repeat-containing protein, [*Cystobacter ferrugineus*], WP_071903904.1 | 46 |
| 2 | 2583 | Lap B | NRPS | Non-ribosomal peptide synthase [*Cystobacter ferrugineus*], WP_071903896.1 | 52 |
| 3 | 1878 | Lap C | Iterative methylation of PABA | radical SAM protein [*Corallococcus sp. CA054B*],  RKG65320.1 | 69 |
| 4 | 1179 | Lap D | Dioxygenation of PABA | benzoyl-CoA 2,3-epoxidase subunit BoxB [*Myxococcales bacterium*], RYZ03280.1 | 50 |
| 5 | 480 | Lap E | Unknown function | DUF1579 domain-containing protein [*Mizugakiibacter sediminis*], GAP65529.1 | 42 |
| 6 | 957 | Lap F | Hydroxylation of PABA | alpha/beta hydrolase [*Corallococcus sp. CA054B*]_WP_120590331.1 | 43 |
| 7 | 1038 | Lap G | Methylation of PABA | methyltransferase domain-containing protein [*Cystobacter ferrugineus*], WP_120590334.1 | 61 |
| 8 | 5913 | Lap H | NRPS | non-ribosomal peptide synthetase [*Cystobacter ferrugineus*], WP_071903897.1 | 46 |
| 9 | 1191 | Lap I | Thiazole ring biosynthesis | flavin-dependent dehydrogenase [*Myxococcales bacterium*], TFH32671.1 | 45 |
| 10 | 801 | Lap J | Type II thioesterase | Thioesterase [*Alteromonadaceae bacterium*], NQY65012.1 | 46 |
| 11 | 16311 | Lap K | NRPS | Non-ribosomal peptide synthase [*Corallococcus sp. CA031C*], WP_120582308.1 | 35 |
| 12 | 2097 | Lap L | NRPS | Non-ribosomal peptide synthase/polyketide synthtase [*Cystobacter sp. Cbv34*], QQZ45557.1 | 54 |
| 13 | 9465 | Lap M | NRPS | Non-ribosomal peptide synthase/polyketide synthase [*Thalassomonas actiniarum*], WP_044833293.1 | 37 |
| 14 | 204 | Lap N | Unknown NRPS function | MbtH family NRPS accessory protein [*Cystobacter fuscus*], WP_095990516.1 | 73 |
| 15 | 897 | Lap O | PABA biosynthesis | Branched-chain amino acid aminotransferase [*Lysobacter enzymogenes*], WP_168356146.1 | 45 |
| 16 | 2193 | Lap P | PABA biosynthesis | Aminodeoxychorismate synthase component I [*Lysobacter antibioticus*], WP_152566132.1 | 51 |

**Supplementary Table 2. Gene table for all ORFs within 10kb of the predicted *lap* BGC boundaries.**

| ORF | Gene size (bp) | Protein, [Source Organism], Accession number | ID % |
| --- | --- | --- | --- |
| Upstream |  |  |  |
| -1 | 438 | AAA family ATPase [Armatimonadetes bacterium], HFO60708.1 | 91% |
| -2 | 201 | Site-specific integrase [Armatimonadetes bacterium], HFO60057.1 | 65% |
| -3 | 297 | Site-specific integrase [Armatimonadetes bacterium], PYR16803.1 | 78% |
| -4 | 174 | No significant similarity found | N/A |
| -5 | 582 | Hypothetical protein [Candidatus Aminicenantes bacterium], MBN1225059.1 | 38% |
| -6 | 213 | Hypothetical protein [Acidobacteria bacterium SCN 69-37], ODS55132.1 | 41% |
| -7 | 2889 | AAA family ATPase [Thermoanaerobaculia bacterium], MBZ0111668.1 | 89% |
| -8 | 138 | Hypothetical protein [Acidobactera bacterium], QFW20463.1 | 48% |
| -9 | 450 | recombinase [Vicinamibacteria bacterium], BCS35026.1 | 32% |
| -10 | 261 | Hypothetical protein [Luteitalea sp.], MPZ17527.1 | 73% |
| Downstream |  |  |  |
| 1-1 | 414 | No significant similarity found | N/A |
| 1-2 | 420 | No significant similarity found | N/A |
| 1-3 | 786 | 4’-phophopantetheinyl transferase, [Acidobacteria bacterium], PYQ77455.1 | 45% |
| 1-4 | 306 | No significant similarity found | N/A |
| 1-5 | 234 | No significant similarity found | N/A |
| 1-6 | 228 | IS3 family transpose [Proteobacteria bacterium], KAA3651106.1 | 62% |
| 1-7 | 324 | Transpose [Acidobacteria bacterium], MBA3639762.1 | 71% |
| 1-8 | 471 | Transpose [Burkholderia cepacia], KWO11900.1 | 46% |
| 1-9 | 672 | Hypothetical protein DMG02_00920, [Acidobacteria bacterium], PYQ92574 | 33% |
| 1-10 | 681 | Crp/Fnr family transcriptional regulator [Acidobacteria bacterium], MBV9211192.1 | 46% |
| 1-11 | 492 | DNA repair protein RadC [Luteitalea sp.] | 63% |
| 1-12 | 246 | Hypothetical protein [Acidobacteria bacterium], MBW8866329.1 | 43% |

**Supplementary Table 3. Module homology analysis of lapcin BGC.**

| A domain substrate | Module number | Domains | Protein, [Source Organism], Accession number | Coverage % | ID % | Related NPs |
| --- | --- | --- | --- | --- | --- | --- |
| Ala | Module 1 | CAT | amino acid adenylation domain-containing protein [*Rhodanobacteraceae bacterium*], MBL8300792.1 | 99 | 43 | Uncharacterized BGC |
| Ser | Module 2 | CAT | amino acid adenylation domain-containing protein [*Gemmatimonadetes bacterium*], MBV9774839.1 | 99 | 45 | Uncharacterized BGC |
| Ala | Module 3 | CAT | non-ribosomal peptide synthetase [*Pyxidicoccus sp. SCPEA002*], WP_206727927.1 | 100 | 44 | Uncharacterized BGC |
| PABA | Module 4 | CAT | non-ribosomal peptide synthase [*Cystobacter sp. Cbv34*], AKP45395.1 | 100 | 52 | Cystobactamid |
| Cys | Module 5 | CAT | non-ribosomal peptide synthetase [*Pseudoalteromonas luteoviolacea*], WP_065792831.1 | 100 | 51 | Uncharacterized BGC |
| Cys | Module 6 | CAT | amino acid adenylation domain-containing protein [*Streptomyces kasugaensis*], TBO60313.1 | 99 | 52 | Uncharacterized BGC |
| Asn | Module 7 | AT | non-ribosomal peptide synthase [*Cystobacter sp. Cbv34*], AKP45395.1 | 99 | 52 | Cystobactamid |
| PABA | Module 8 | CAT | non-ribosomal peptide synthase [*Cystobacter sp. Cbv34*], AKP45395.1 | 98 | 50 | Cystobactamid |
| AHIBA | Module 9 | AT | AMP-binding protein [*Xanthomonas albilineans*], WP_045761541.1 | 100 | 52 | Albicidin |
| PABA | Module 10 | CAT | non-ribosomal peptide synthase [*Cystobacter sp. Cbv34*], AKP45395.1 | 99 | 44 | Cystobactamid |

**Supplementary Table 4: MIC values of lapcin against bacteria and fungi**

| Name | Strain | Medium | MIC (µg/mL) | |
| --- | --- | --- | --- | --- |
|  |  |  | Lapcin | Ciprofloxacin |
| *Escherichia coli* | ATCC25922 | LB^a^ | > 32 | < 0.03 |
| *Enterococcus faecium* | Com15 | LB | > 32 | 0.5 |
| *Staphylococcus aureus* | SH1000 | LB | > 32 | 0.5 |
| *Klebsiella pneumonia* | ATCC10031 | LB | > 32 | < 0.03 |
| *Acinetobacter baumannii* | ATCC17978 | LB | > 32 | 0.125 |
| *Pseudomonas aeruginosa* | PAO1 | LB | > 32 | 0.125 |
| *Enterobacter cloacae* | ATCC13047 | LB | > 32 | 0.06 |
| *Candida albicans* | ATCC76485 | YPD^b^ | > 32 | > 16 |

MIC values were measured in duplicate (n=2). ^a^ Luria Bertani broth, ^b^ Yeast Extract Peptone Dextrose.

**Supplementary Table 5: Cell line information**

| Cell Line | Cell Panel | Culture Medium | Inoculation Density (cells/well)^a^ |
| --- | --- | --- | --- |
| HT29 | Colon | DMEM + 10% FBS | 2,500 |
| Colo205 | Colon | RPMI-1640 + 10% FBS | 2,500 |
| HCT116 | Colon | DMEM + 10% FBS | 10,000 |
| SW480 | Colon | DMEM + 10% FBS | 10,000 |
| MCF7 | Breast | DMEM + 10% FBS | 8,000 |
| HCC1806 | Breast | RPMI-1640 + 10% FBS | 2,500 |
| A549 | Lung | DMEM + 10% FBS + NEAA | 2,500 |
| NCI-H1299 | Lung | RPMI-1640 + 10% FBS | 2,500 |
| NCI-H226 | Lung | RPMI-1640 + 10% FBS | 10,000 |
| Hela | Cervical | DMEM + 10% FBS | 2,500 |
| U2OS | Bone | DMEM + 10% FBS + NEAA | 2,500 |
| HEK293 | Normal cell | DMEM + 10% FBS | 5, 000 |

^a^Cell numbers were counted by a Countess® II FL Automated Cell Counter.

**Supplementary Table 6. List of PCR primers used in this study.**

| Primer Name | Sequence | Function |
| --- | --- | --- |
| NRPS-A-FW | GCSTACSYSATSTACACSTCSGG | NRPS A domain screening |
| NRPS-A-RV | SASGTCVCCSGTSCGGTA | NRPS A domain screening |
| P30-w539-FW | ATGTACACGTCGGGTTCCAC | Clone recovery of cosmid DFD000327-539 |
| P30-w539-RV | AGCAGCTCTTTCACCGAGAT | Clone recovery of cosmid DFD000327-539 |
| P30-w11-FW | GGCCAGCATGTATTCGATCT | Clone recovery of cosmid DFD000327-11 |
| P30-w11-RV | AGTCTGCGCCTCTTCATGTT | Clone recovery of cosmid DFD000327-11 |
| P30-539-UPS-FW02 | ATCTCGAGGCTATTAGAGATCGAAAGGGACGTTCA | Amplification of Upstream homology arm for TAR |
| P30-539-UPS-RV02 | CGAAGAAGTGAAGAGGGACGGTTTAAACTCTCTCCGCAATGAGATCCT | Amplification of Upstream homology arm for TAR |
| P30-11-DWS-FW02 | GTTTAAACCGTCCCTCTTCACTTCTTCG | Amplification of Downstream homology arm for TAR |
| P30-11-DWS-RV02 | GTACCGAGCTCGTTACCTGCAAGACCATCGCTAAT | Amplification of Downstream homology arm for TAR |
| P30-SC-1F | CGACCCACTCGAGCTCTATC | Screening primers for fidelity of TAR |
| P30-SC-1R | CCCAACGCACCAGAGTAAAT | Screening primers for fidelity of TAR |
| P30-SC-2F | GTCGTTAGCAGCTCCACCTC | Screening primers for fidelity of TAR |
| P30-SC-2R | ACGTGATCGGATTTTTCGTC | Screening primers for fidelity of TAR |
| P30-SC-3F | TTCATCAGTTCAGGCAGTCG | Screening primers for fidelity of TAR |
| P30-SC-3R | ACCTCTCGGATTCGTTCCTT | Screening primers for fidelity of TAR |
| P30-SC-4F | CCTCGCGTACTGCATCTACA | Screening primers for fidelity of TAR |
| P30-SC-4R | AGCAGGTAGACGCGAGTGTT | Screening primers for fidelity of TAR |
| IRL | ACAGGTTGGCTGATAAGTCCCCGGTCT | inverted repeat sequence for pTARa4 |
| IRR | AGACCGGGGACTTATCAGC CAACCTGT | inverted repeat sequence for pTARa4 |


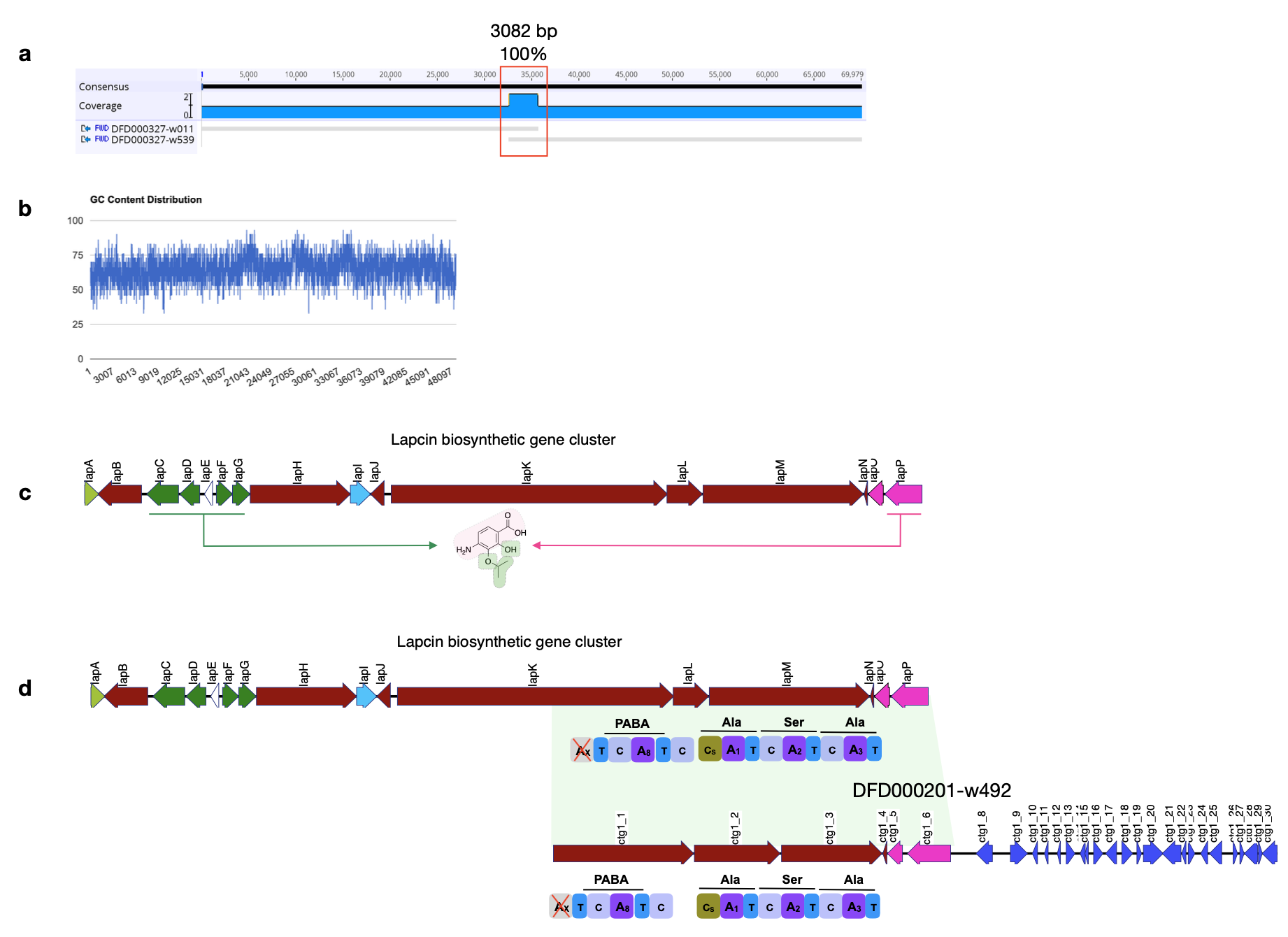
**Supplementary Figure 1. The lap BGC occurs naturally.** Diverse analyses indicate that the lap BGC occurs naturally and was not generated as an artificial fusion of gene fragments during the cloning process. a) Overlapping cosmid clones. The lapcin BGC was captured on two overlapping metagenomic cosmid clones: DF000327-w011 and DFD000327-W539. The overlapping region spans 3082 bp and there is 100% identity between the two clones. b) Lap BGC GC content. We did not see any abrupt changes in GC content or obviously truncated genes that would suggest the lap BGC is an artificial fusion from the cloning process. c) Overview of the position of PABA biosynthesis genes in the lap BGC. The biosynthesis of PABA requires 6 distinct genes. PABA biosynthesis genes are found on both sides of the lap BGC. Both overlapping clones are needed to have the full complement of PABA biosynthetic genes. The chance that clones from two unrelated BGCs were randomly fused to reconstitute the full complement of genes needed for PABA biosynthesis is very unlikely. d) Analysis of a cosmid clone containing a second lap BGC that was recovered from a distinct soil metagenomic library indicate that the PABA and proteinogenic amino acid halves of the lap BGC were not artificially fused. We recovered the PABA/proteinogenic amino acid fusion region of the lap BGC from a completely independent metagenomic library (i.e., a library that was constructed from a different soil at a different time). Although this second copy of the lap BGC differs slightly by DNA sequence, it contains the exact same gene content, gene organization, NPRS domain content and NRPS domain organization as does the original lap BGC we recovered.


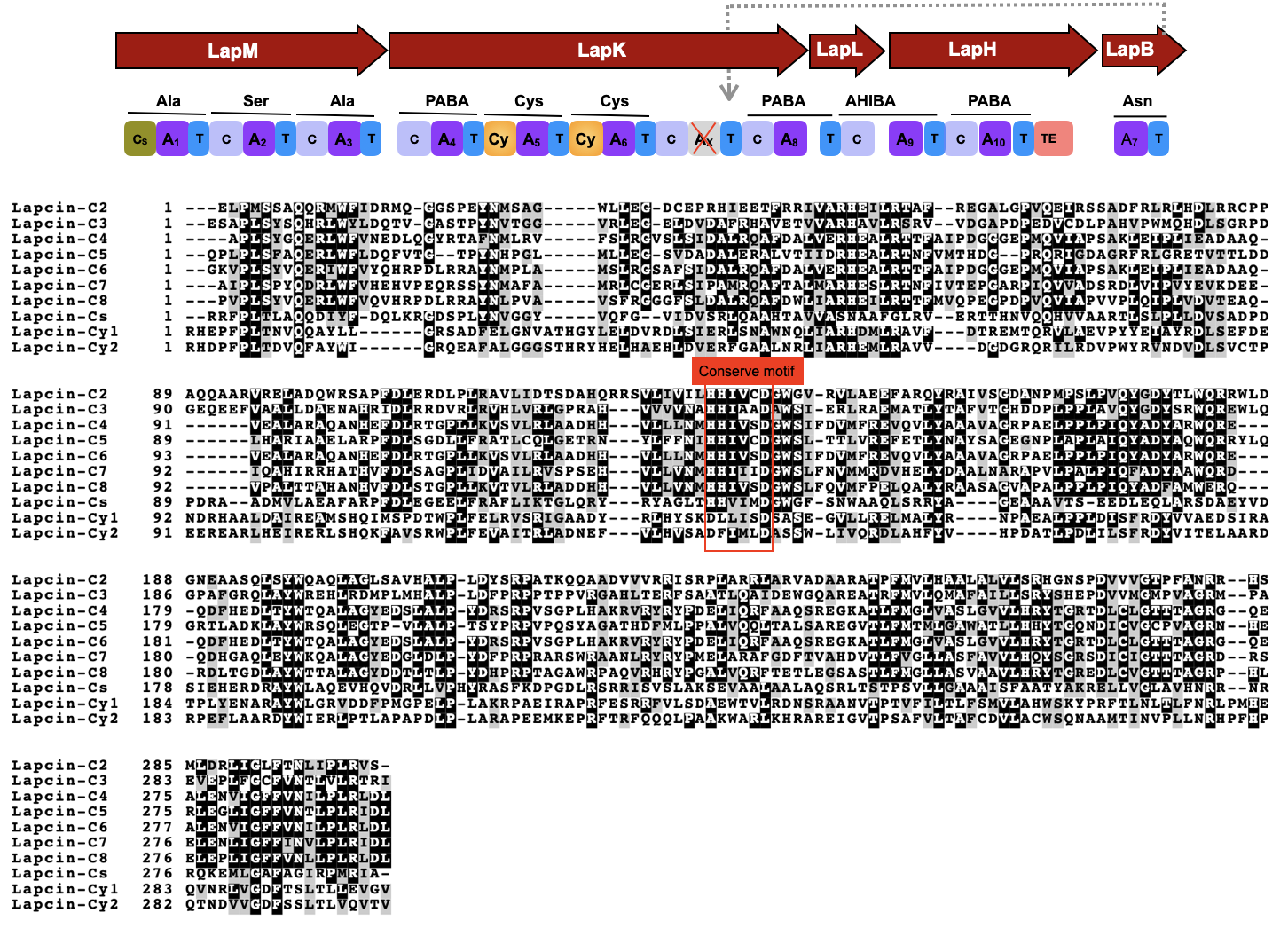


**Supplementary Figure 2:** Sequence alignment of lap BGC condensation (C) domains. Heterocyclization domains (Cy1 and Cy2) have a conserved DXXXXD motif in the place of the conserved HHXXXD motif seen in other condensation domains.


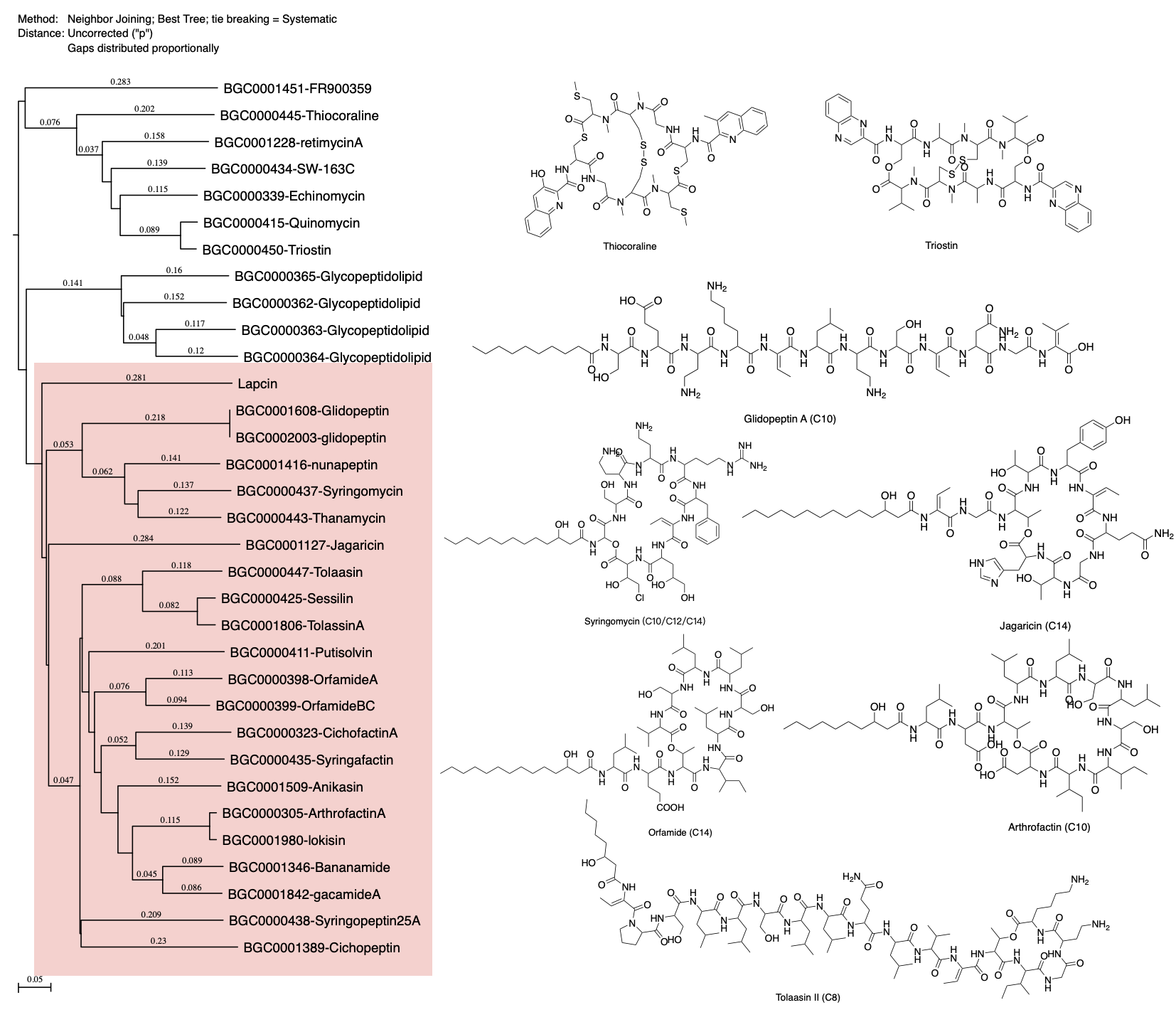


**Supplementary Figure 3:** Phylogenetic tree of C_s_ domain sequences. The lap BGC starter domain is most closely related to Cs domain sequences that use 3-hydroxy substituted C10-C14 fatty acids as substrate (red box). Lapcin was therefore designed with an N-terminal 3-hydroxymyristic acid.


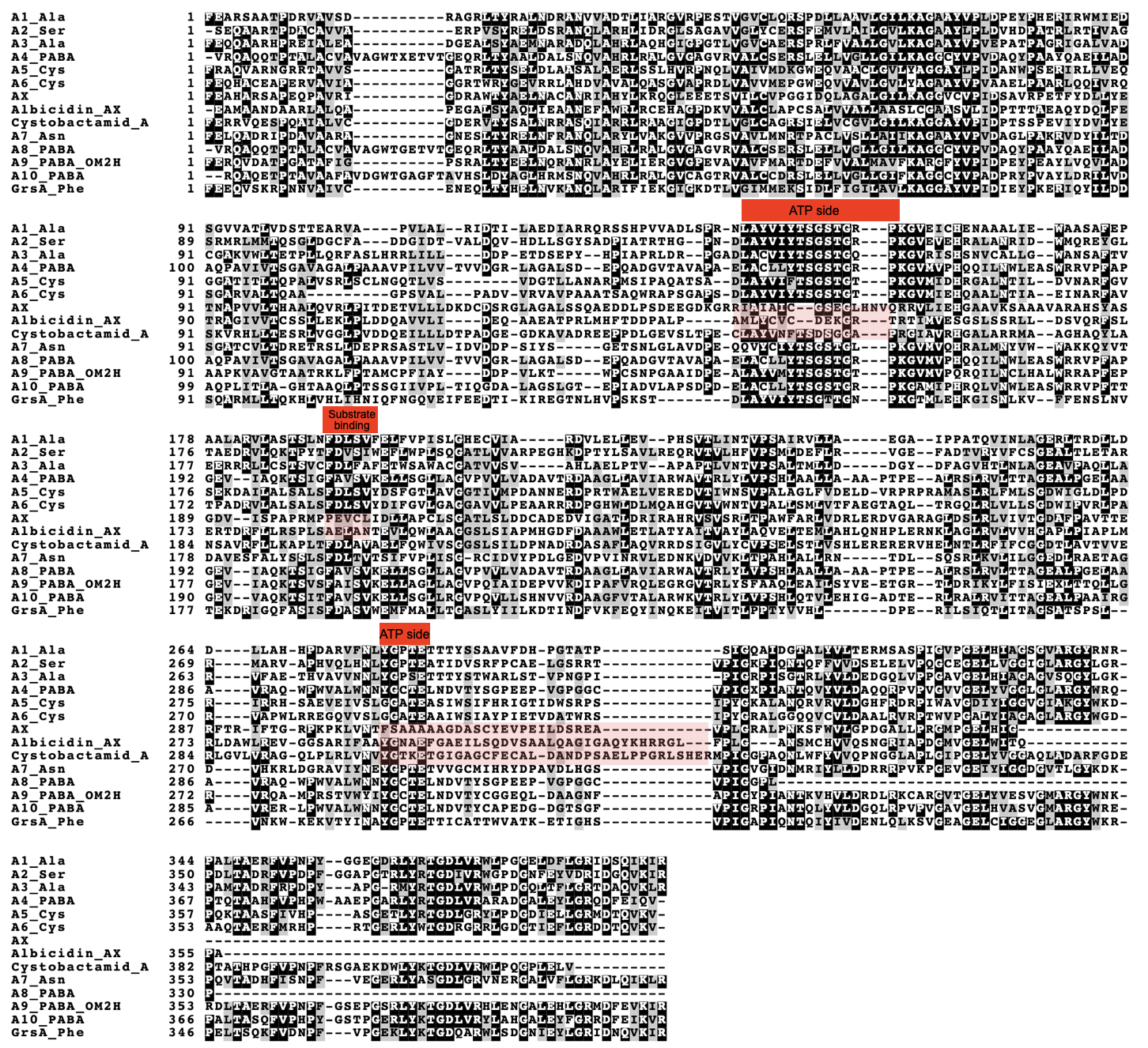


**Supplementary Figure 4:** Sequence alignment of lap BGC NRPS A-domains. The alignment shows several variations in the LapK Ax domain (ATP, Mg^2+^ and substrate binding sites) indicating that this domain is likely inactive. Similar sequence variations are seen in nonfunctional A-domains found in other PABA-encoding BGCs (i.e., Albicidin and Cystobactamid BGCs). As seen in other PABA-encoding BGCs, the LapB A-domain is predicted to compensate for this inactive A-domain.

**Supplementary Figure 5.** Solid phase synthesis of intermediate 1, Fragment A.

**Supplementary Figure 6:** Synthetic pathway for the preparation of intermediate **7**, Fragment B.

**Supplementary Figure 7:** Synthetic pathway for the preparation of intermediate **16**, Fragment C.

**Supplementary Figure 8:** Final coupling pathway for **lapcin**.

 **Supplementary Figure 9.** ^1^H NMR (600MHz, MeOD-*d_4_*) spectrum of **1**

**Supplementary Figure 10.** ^1^H NMR (600MHz, DMSO-*d_6_*) spectrum of **3**

**Supplementary Figure 11.** ^1^H NMR (600MHz, DMSO-*d_6_*) spectrum of **4**

**Supplementary Figure 12.** ^1^H NMR (600MHz, DMSO-*d_6_*) spectrum of **5**

**Supplementary Figure 13.** ^1^H NMR (600MHz, DMSO-*d_6_*) spectrum of **6**

**Supplementary Figure 14.** ^1^H NMR (600MHz, MeOD-*d_4_*) spectrum of **7**

**Supplementary Figure 15.** ^1^H NMR (400MHz, CDCl_3_) spectrum of **9**

**Supplementary Figure 16.** ^1^H NMR (400MHz, CDCl_3_) spectrum of **10**

**Supplementary Figure 17.** ^1^H NMR (400MHz, CDCl_3_) spectrum of **11**

**Supplementary Figure 18.** ^1^H NMR (400MHz, CDCl_3_) spectrum of **12**

**Supplementary Figure 19.** ^1^H NMR (400MHz, CDCl_3_) spectrum of **13**

**Supplementary Figure 20.** ^1^H NMR (400MHz, DMSO-*d_6_*) spectrum of **14**

**Supplementary Figure 21.** ^1^H NMR (400MHz, DMSO-*d_6_*) spectrum of **15**

**Supplementary Figure 22.** ^1^H NMR (600MHz, MeOD-*d_4_*) spectrum of **S15-1**

**Supplementary Figure 23.** ^1^H NMR (600MHz, MeOD-*d_4_*) spectrum of **16**

**Supplementary Figure 24.** ^1^H NMR (600MHz, DMSO-*d_6_*) spectrum of **S17**

**Supplementary Figure 25.** ^1^H NMR (400MHz, DMSO-*d_6_*) spectrum of **S19**

**Supplementary Figure 26.** ^1^H NMR (600 MHz, DMSO-*d_6_*) spectrum of **lapcin**

**Supplementary Figure 27.** ^13^C NMR (400 MHz, DMSO-*d_6_*) spectrum of **lapcin**

**Supplementary Figure 28.** ^1^H-^13^C HSQC NMR spectrum of **lapcin**


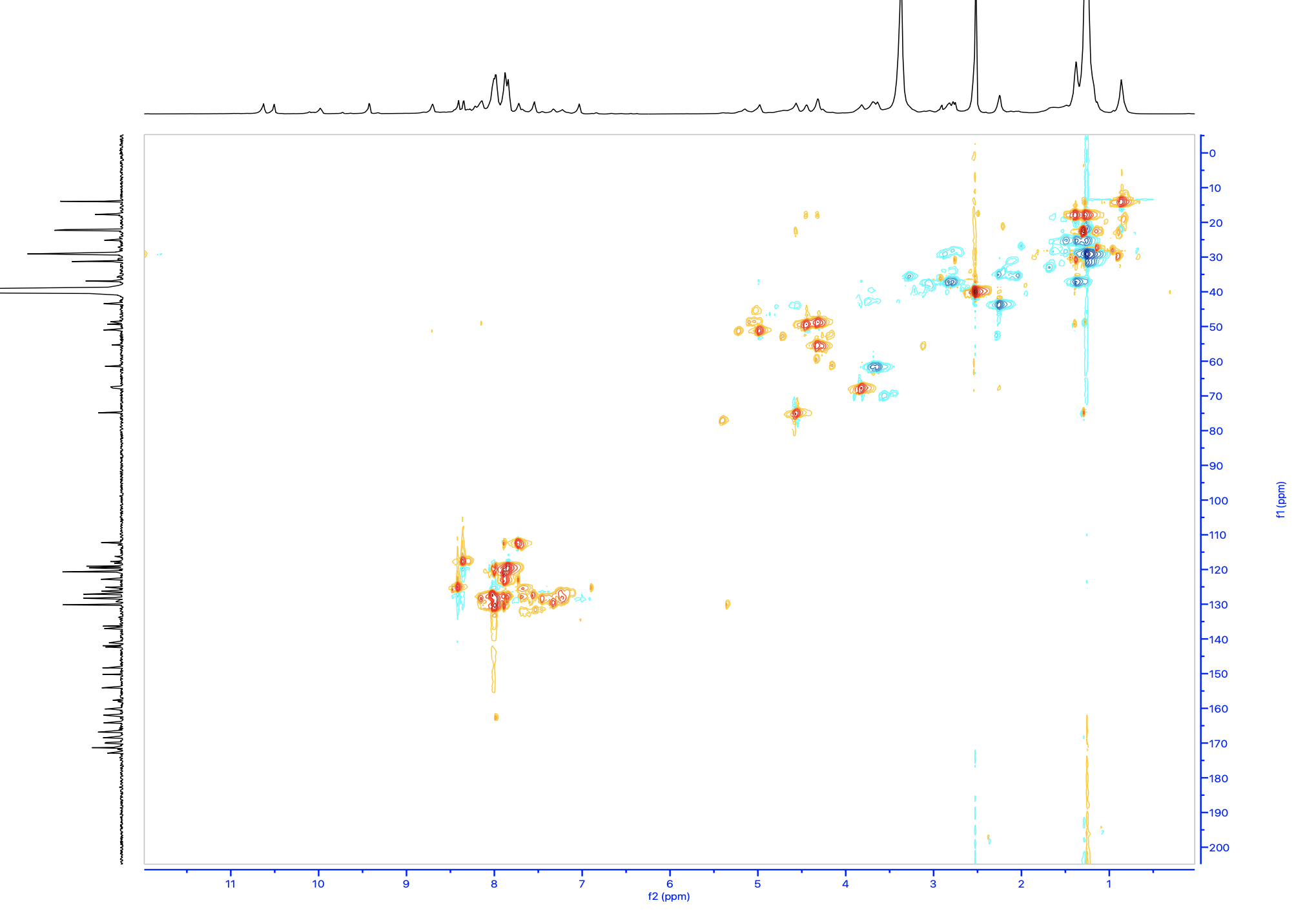


**Supplementary Figure 29.** ^1^H-^13^C HMBC NMR spectrum of **lapcin**

**
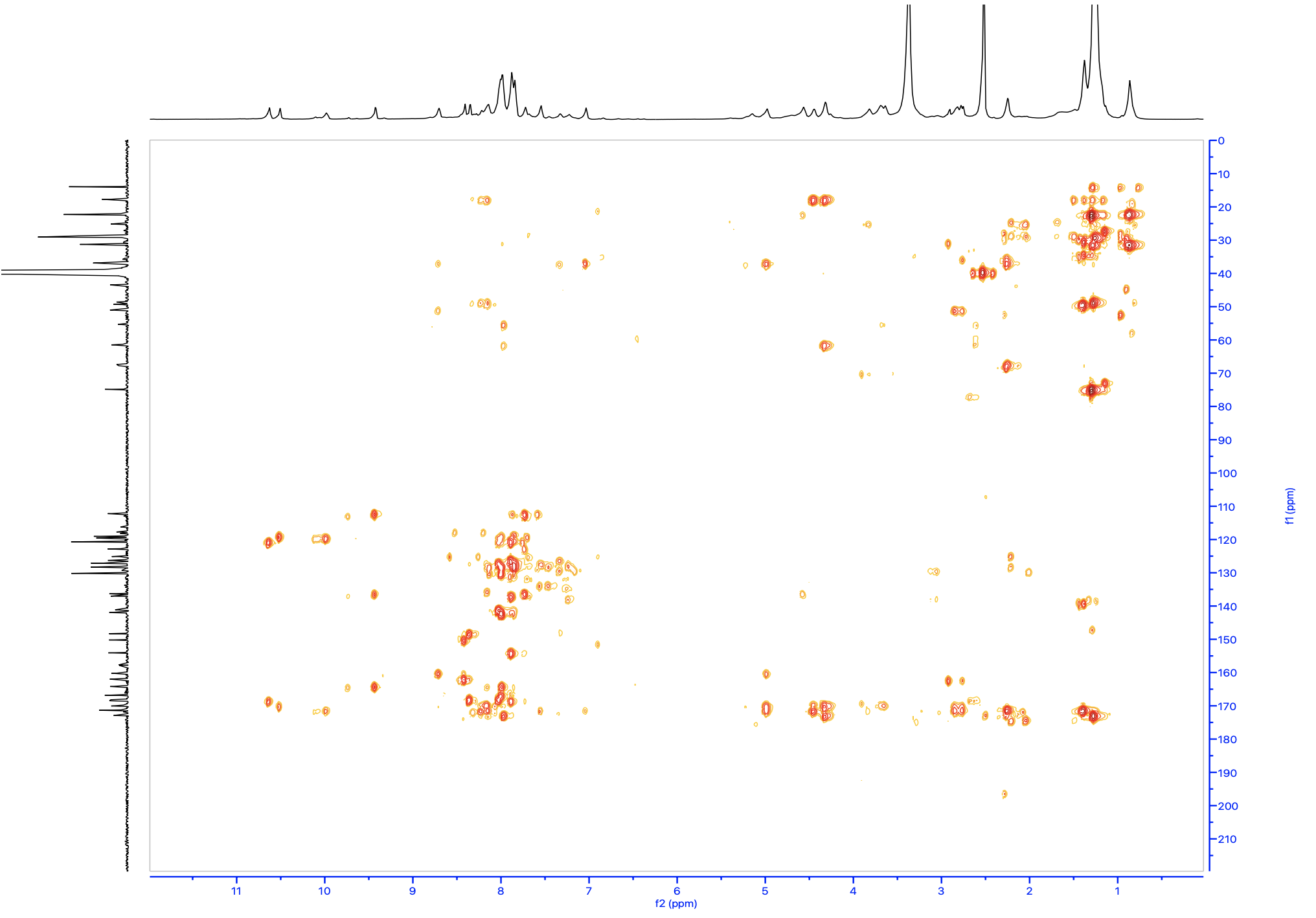
**

**Supplementary Figure 30.** ^1^H-^1^H COSY NMR spectrum of **lapcin**

**
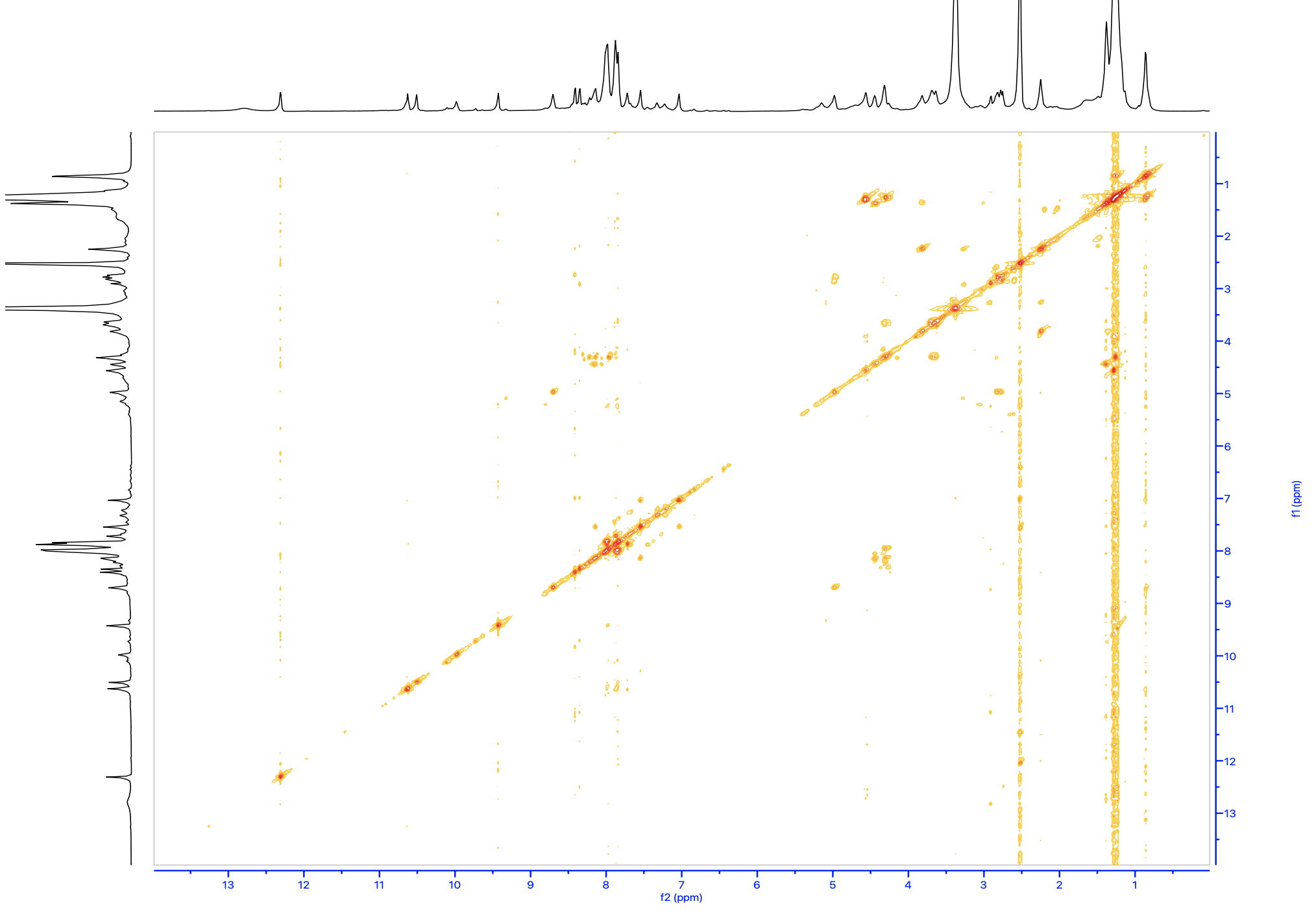
**


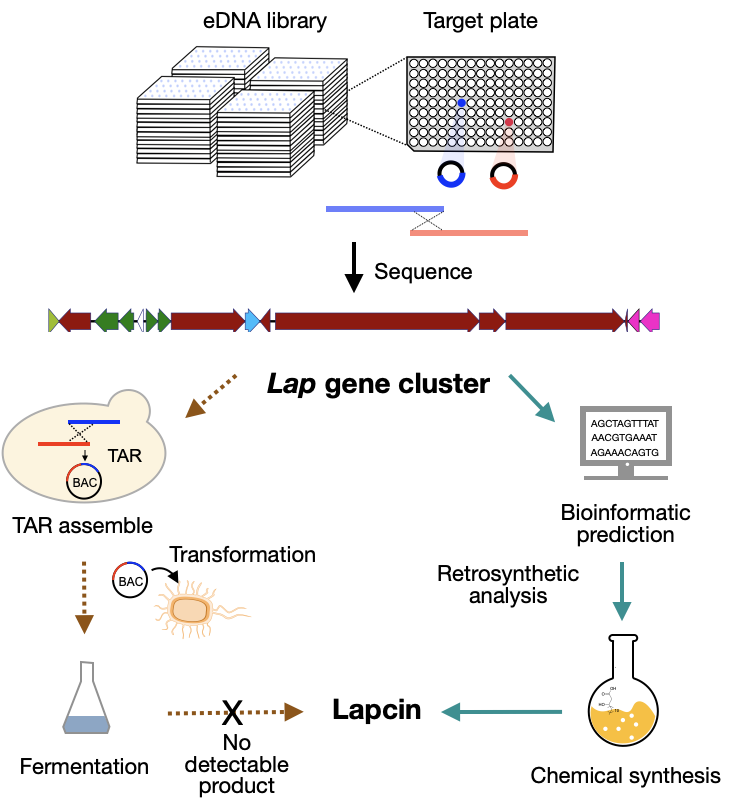


**Supplementary Figure 31.** Summary of methods attempted to access lapcin bioactive molecules. Left panel, traditional heterologous expression, right panel: bioinformatic guided total chemical synthesis.


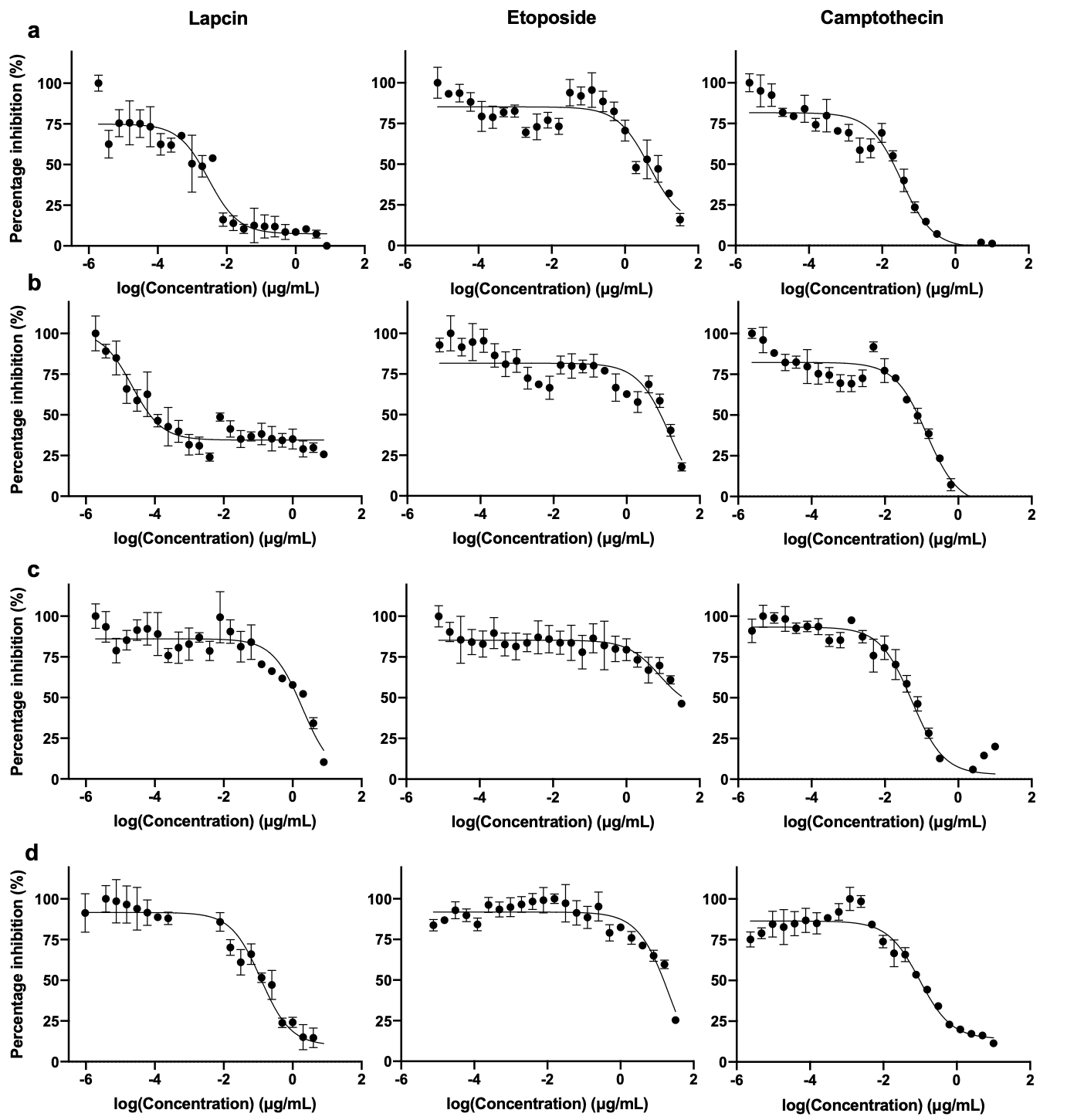


**Supplementary Figure 32**: IC50 curves for colon cancer cell lines. a) HT29 cell line, b) colo205 cell line, c) HCT116 cell line, d) SW480 cell line. N=3 biological independent assays, mean value and SD are shown.


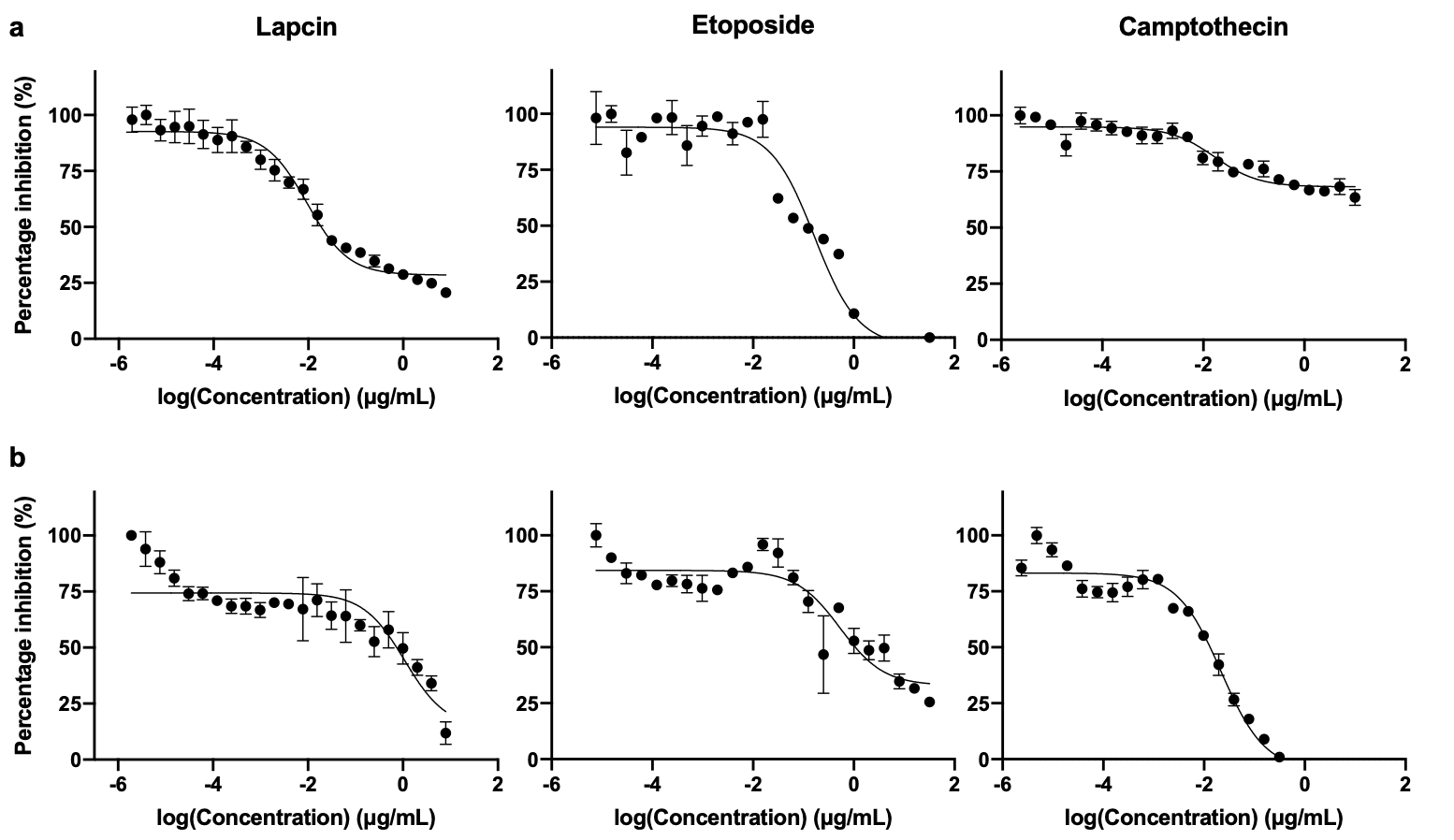


**Supplementary Figure 33**: IC50 curves for breast cancer cell line. a) MCF7 cell line, b) HCC1806 cell line. N=3 biological independent assays, mean value and SD are shown.


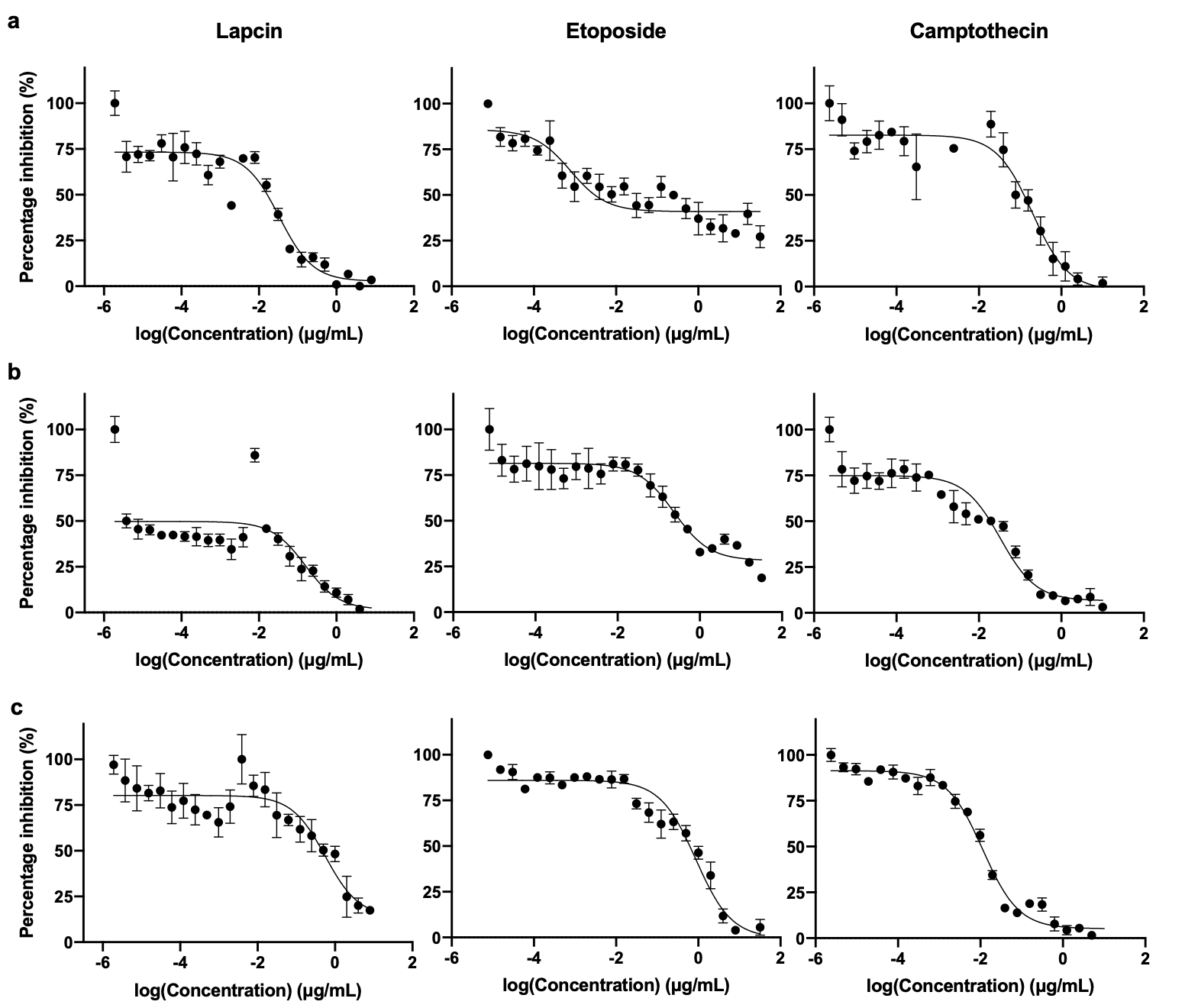


**Supplementary Figure 34**: IC50 curves for Lung cancer cell line. a) A549 cell line, b) NCI-H1299 cell line, c) NCI-H226 cell line. N=3 biological independent assays, mean value and SD are shown.


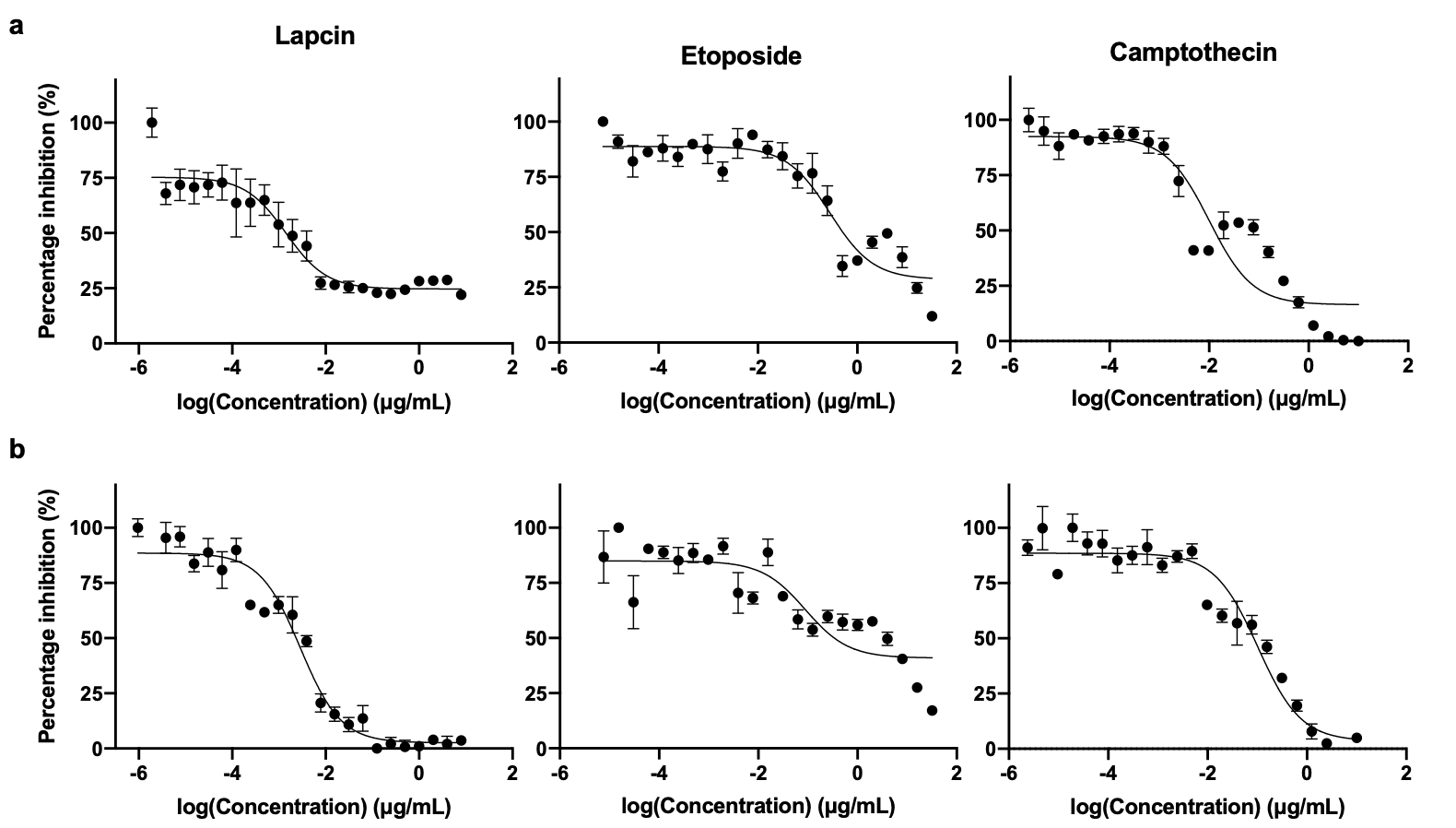


**Supplementary Figure 35**: IC50 curves for other cancer cell lines. a) Hela cell line, b) U2OS cell line. N=3 biological independent assays, mean value and SD are shown.


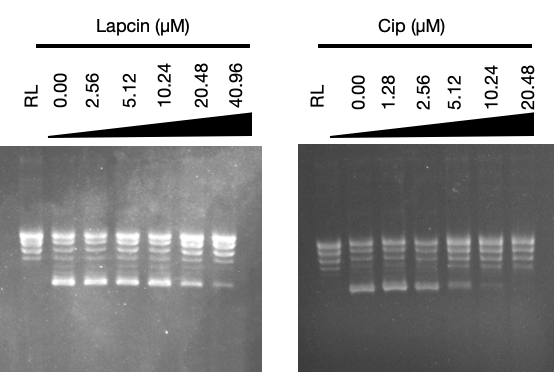


**Supplementary Figure 36**: DNA gyrase supercoiling assay with Lapcin or ciprofloxacin (CIP) at the concentrations indicated (RL, relaxed plasmid).

**
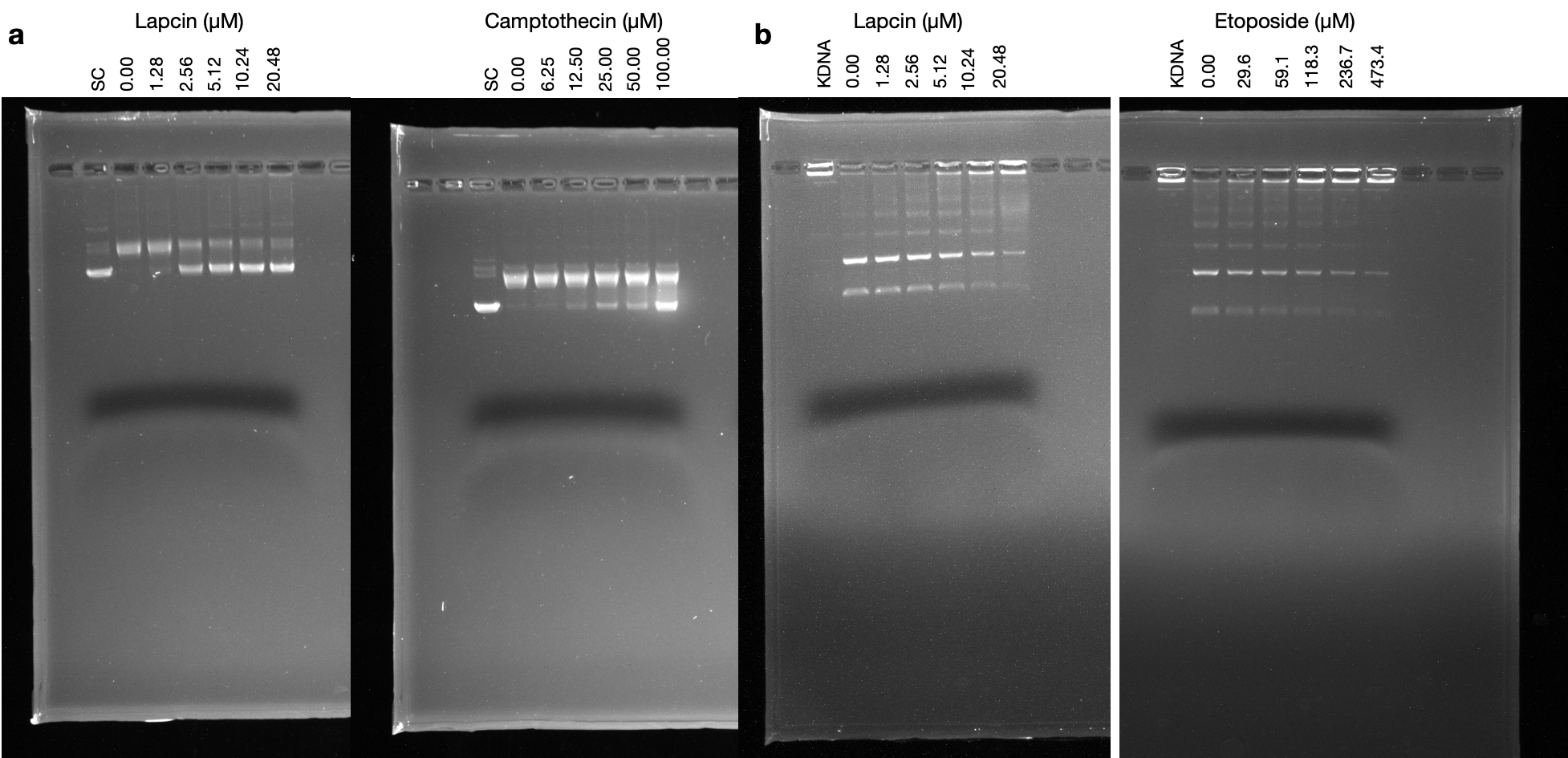
**

**Supplementary Figure 37.** Uncropped and unprocessed scans of topoisomerase inhibition gels. a) Type I topoisomerase DNA relaxation assay. b) Type II topoisomerase DNA decatenation assay.


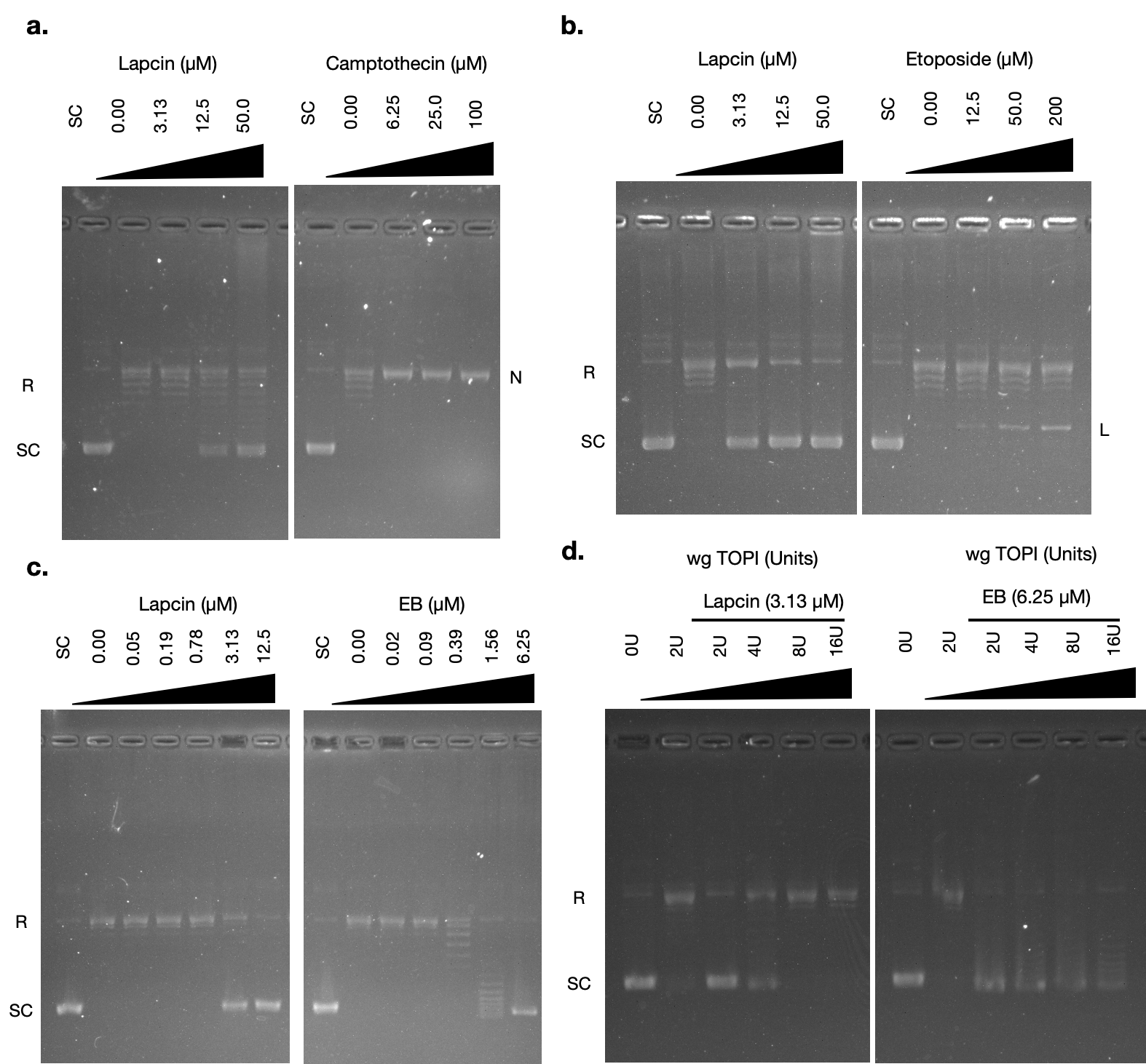


**Supplementary Figure 38.** Characterization of lapcin’s topoisomerase inhibition activity. a) Type I topoisomerase DNA cleavage assay. b) Type II topoisomerase DNA cleavage assay. c) DNA interaction assay with a fixed amount of wheat germ topoisomerase I. d) DNA intercalation assay using a fixed concentration of lapcin or EB. relaxed DNA (R), supercoiled DNA (SC), linear DNA (L), Open nick DNA (N). Those assays were tested two independent times. Both of them showed similar results. Representative gel picture was shown.

**Supplementary References:**

1 Owen, J. G. *et al.* Multiplexed metagenome mining using short DNA sequence tags facilitates targeted discovery of epoxyketone proteasome inhibitors. *Proc Natl Acad Sci U S A* **112**, 4221-4226, doi:10.1073/pnas.1501124112 (2015).

2 Kim, J. H. *et al.* Cloning large natural product gene clusters from the environment: piecing environmental DNA gene clusters back together with TAR. *Biopolymers* **93**, 833-844, doi:10.1002/bip.21450 (2010).

3 Fu, J. *et al.* Efficient transfer of two large secondary metabolite pathway gene clusters into heterologous hosts by transposition. *Nucleic Acids Res* **36**, e113, doi:10.1093/nar/gkn499 (2008).

4 Kallifidas, D. & Brady, S. F. Reassembly of functionally intact environmental DNA-derived biosynthetic gene clusters. *Methods Enzymol.* **517**, 225-239, doi:10.1016/B978-0-12-404634-4.00011-5 (2012).

5 Hover, B. M. *et al.* Culture-independent discovery of the malacidins as calcium-dependent antibiotics with activity against multidrug-resistant Gram-positive pathogens. *Nat Microbiol* **3**, 415-422, doi:10.1038/s41564-018-0110-1 (2018).

6 Wu, C., Shang, Z., Lemetre, C., Ternei, M. A. & Brady, S. F. Cadasides, Calcium-Dependent Acidic Lipopeptides from the Soil Metagenome That Are Active against Multidrug-Resistant Bacteria. *J. Am. Chem. Soc.* **141**, 3910-3919, doi:10.1021/jacs.8b12087 (2019).

7 NCCLS/CLSI. (NCCLS/CLSI Wayne, 2003).
